# Methylation profiles of thirty four promoter-CpG islands and concordant methylation behaviours of sixteen genes that may contribute to carcinogenesis of astrocytoma

**DOI:** 10.1186/1471-2407-4-65

**Published:** 2004-09-14

**Authors:** Jian Yu, Hongyu Zhang, Jun Gu, Song Lin, Junhua Li, Wei Lu, Yifei Wang, Jingde Zhu

**Affiliations:** 1 Cancer Epigenetics and Gene Therapy, State-Key Laboratory for Oncogenes and Related Genes, Shanghai Cancer Institute, Shanghai Jiao Tong University, LN 2200/25, Xie-Tu Road, Shanghai 200032, China; 2 Department of Neurosurgery, Tiantan Hospital of Capital University of Medical Sciences, Beijing Neurosurgical Institute, Beijing 100050, China; 3 Department of Mathematics, Shanghai University, No. 99, Shangda Road, Shanghai 200436, P. R. China

## Abstract

**Background:**

Astrocytoma is a common aggressive intracranial tumor and presents a formidable challenge in the clinic. Association of altered DNA methylation patterns of the promoter CpG islands with the expression profile of  cancer-related genes, has been found in many human tumors. Therefore, DNA methylation status as such may serve as an epigenetic biomarker for both diagnosis and prognosis of human tumors, including astrocytoma.

**Methods:**

We used the methylation specific PCR in conjunction with sequencing verification to establish the methylation profile of the promoter CpG island of thirty four genes in astrocytoma tissues from fifty three patients (The WHO grading:. I: 14, II: 15, III: 12 and IV: 12 cases, respectively). In addition, compatible tissues (normal tissues distant from lesion) from three non-astrocytoma patients were included as the control.

**Results:**

Seventeen genes (*ABL, APC, APAF1, BRCA1, CSPG2, DAPK1, hMLH1, LKB1, PTEN, p14^ARF^, p15^INK4b^, p27^KIP1^, p57^KIP2^, RASSF1C, RB1, SURVIVIN*, and *VHL*) displayed a uniformly unmethylated pattern in all the astrocytoma and non-astrocytoma tissues examined. However, the *MAGEA1 *gene that was inactivated and hypermethylated in non-astrocytoma tissues, was partially demethylated in 24.5% of the astrocytoma tissues (co-existence of the hypermethylated and demethylated alleles). Of the astrocytoma associated hypermethylated genes, the methylation pattern of the *CDH13, cyclin a1, DBCCR1, EPO, MYOD1*, and *p16*^*INK4a *^genes changed in no more than 5.66% (3/53) of astrocytoma tissues compared to non-astrocytoma controls, while the *RASSF1A, p73, AR, MGMT, CDH1, OCT6,, MT1A, WT1*, and *IRF7 *genes were more frequently hypermethylated in 69.8%, 47.2%, 41.5%, 35.8%, 32%, 30.2%, 30.2%, 30.2% and 26.4% of astrocytoma tissues, respectively. Demethylation mediated inducible expression of the *CDH13, MAGEA1, MGMT, p73 *and *RASSF1A *genes was established in an astrocytoma cell line (U251), demonstrating that expression of these genes is likely regulated by DNA methylation. *AR gene *hypermethylation was found exclusively in female patients (22/27, 81%, 0/26, 0%, P < 0.001), while the *IRF7 gene *hypermethylation preferentially occurred in the male counterparts (11/26, 42.3% to 3/27, 11%, P < 0.05). Applying the mathematic method "the Discovery of Association Rules", we have identified groups consisting of up to three genes that more likely display the altered methylation patterns in concert in astrocytoma.

**Conclusions:**

Of the thirty four genes examined, sixteen genes exhibited astrocytoma associated changes in the methylation profile. In addition to the possible pathological significance, the established concordant methylation profiles of the subsets consisting of two to three target genes may provide useful clues to the development of the useful prognostic as well as diagnostic assays for astrocytoma.

## Background

Diffusely infiltrating astrocytoma is a leading group of the primary central nervous system tumors, accounting for more than 60% of all primary brain tumors [1,2]. It may arise aggressively from the normal astrocytes, or evolve stepwise from the less its benign precursors. Owing to the difficulties with its early diagnosis and surgical removal of all residue diseased tissues, rapid progression, and frequent reoccurrence, the most advanced form of astrocytoma, glioblastoma (WHO grading IV) represents an extremely life-threatening intracranial malignant tumor both inside and outside of China [1,2]. Molecular genetic analyses have demonstrated multiple genetic lesions implicating to pathogenesis of astrocytoma, glioblastoma in particular. In addition to the frequent amplification and deletion of the EGF receptor gene (*EGFR*) [3], the main genetic events affecting the following tumor suppressor genes: the members of the *INK4A *initiated cell-cycle arrest pathway (the *p16*^*INK4a*^) [4], the *p14*^*ARF *^[5], the *RB1 *[6] and *the p53 *[7]), a wide spectrum of the cell surface receptor genes (i.e., *CD44*, *integrin*, and receptors for various growth factors), and the *PTEN *genes [8].

Transcription in eukaryotes is regulated at multiple levels and inversely correlated with the hypermethylated state as well as the chromatin condensation. It has been well established that the methylation status of CpG islands directly affects the DNA-protein interactions by eliminating the otherwise occurring sequence specific binding of the transcription factors whereas inducing the DNA-bindings of members of the methyl-CpG binding protein family (MBD). Histone modifications (deacetylation and methylation) may occur subsequently leading to chromatin condensation and a long-term transcriptional silencing status of the affected DNA segments. Over 40% of the protein coding genes have at least one CpG island within or near to their promoter, an strong indication for transcription of which is likely to be under the control of DNA methylation status. Three DNA methyl transferases are involved in the control of the methylation state of the CpGs in genome. DNA methyl transferase I is mainly responsible for the maintenance of the methylation status of the genome after DNA replication, while IIIA and IIIB act principally in the *de novo *DNA methylation in the early development of high eukaryotes. DNA methylation patterns in somatic cells are established during the early development and contribute to the allele-specific transcription silencing of the imprinted genes, including the silenced alleles in the X-chromosome and other chromosomes. The epigenetic pattern (the DNA methylation profiles of the genome) in high eukaryotes is integral to the normal execution of the biological activities in cells and needs to be actively maintained. In addition to the changes linked to the cell lineage specific pattern of gene expression, both global hypomethylation and local hypermethylation of the CpG islands occur progressively as cell ages.

Aberrant DNA methylation pattern changes gene transcription that has been etiologically linked to cancer formation [9,10]. The genome-wide hypomethylation has been believed to activate transcription of the otherwise silenced transposon like repetitive sequences (such as the Alu and LINE repeats in mammals). As a result, the transposition occurs more prevalently so that the genomic instability in cancer cells will be significantly increased [11-13]. The hypermethylated state of the promoter CpG islands has been etiologically associated with transcription inactivation of a number of tumor suppressor genes in tumors, which are hypomethylated and transcribed in their normal counterparts. Therefore, the hypermethylated CpG island(s) of those genes have been regarded as a defect, reminiscent to the loss of heterozygosity or other types of genetic deletion for total inactivation of the tumor suppressor genes in cancer. The most noticeable example is the *p16*^*INK4a *^gene that has been frequently hypermethylated in almost all types of the tumors examined [14-17] including hepatocellular carcinoma [18]. The loss of the genetic imprinting (changes in DNA methylation status) has been found to reactivate transcription of the otherwise silenced allele of the genes such as the insulin like growth factor 2 gene, which has been well documented in human tumors [19]. On the other hand, the reverse process, i.e., demethylation of the promoter CpG island, has also been found instrumental to the transcription activation of the otherwise inert genes in tumor cells [20]. A prominent example is the gene encoding the melanoma antigen, MAGEA1 that was hypermethylated and transcriptionally silenced in the normal liver tissues, and demethylated prevalently in the hepatocellular carcinoma tissues [18], correlating well with the elevated level of its expression in HCC [21,22]. The over-expressed gene, *SURVIVIN*, has also been reported to be demethylated in human ovarian cancer [23]. Despite of the fact that the elevated levels of expression of three DNA methyl transferase genes were detected in virtually all cancers, the profiles of the hypermethylated genes vary with both the types and stages of cancers. Therefore, the undefined defects in the epigenetic homeostasis during carcinogenesis, rather than the aberrant expression of any given DNA methyl transferase, are more likely to account for the cancer type specific pattern of DNA methylation at both global and local levels.

Methylation profiling of the promoter CpG islands has been an important information gathering process for new insights into our understanding of the role of DNA methylation in both initiation and progression of human carcinogenesis. It would result in development of the DNA methylation based assays for cancer diagnosis as well as identification of the cancer genes suffering from the epigenetic defects . However, as the majority of studies had only targeted one or a few genes in rather small patient groups, the concurrent hypermethylation behavior of multiple genes has only been addressed in a limited number of tumor types, such as colorectal cancer. The majority, if not all, of the previous studies on the astrocytoma associated changes in methylation profiles have only examined a small number of genes for methylation status at the promoter CpG island [25,26]. In this study, we determined the methylation profiles of as many as thirty four genes in a cohort consisting of 53 astrocytoma patients and established the concordant methylation behavior of up to three targets. Our observations should provide new insights into the DNA methylation epigenetic defects in human astrocytoma.

## Methods

All the experiments were performed according to protocols described previously [18]. The primer pairs for the methylation specific PCR were either adopted (*APC, BRCA1, CDH1, DAPK1, hMLH1, p14^ARF^, p15^INK4b^, RASSF1A, RB1 *and *VHL*) or designed according to the same principle with assistance of the software packages for the CpG islands identification  and the primer design  [Additional file 1].

### Tissue samples

#### Tissue samples and DNA preparation

With the informed consent of all patients and approval of the ethics committee, the tumor samples were collected from astrocytoma patients (n = 53) during operation at the Tiantan Neurosurgical Hospital in Beijing. The pathological classification of tumor tissues was carried out and the stage of each astrocytoma patients was determined according to the WHO classification [1]. No significant geographic impart was observed as patients came from different places in China and went to Beijing for treatment. In addition, the compatible tissues (normal tissues distant from the lesions) were surgically obtained from three non-astrocytoma patients [gangliocytoma (21 years old, male), angiocavernoma (49 years old, male) and meningioma (54 years old, female)] as the normal controls, which have been subjected to the proper pathological evaluation.

Total genomic DNA was extracted from frozen tissue specimens (50 – 100 mg) according to a standard protocol with some modifications [18,27]. Frozen pulverized powders of the specimens were re-suspended with 2 ml lysis buffer: 50 mM Tris-HCl pH 8.0, 50 mM EDTA, 1% SDS, 10 mM NaCl plus 100 μg/ml boiling-treated RNase A (Sigma). Following one hour of incubation at 37°C, Proteinase K (Roche, USA) was added to the cellular lysates for a final concentration of 100 μg/ml and the digestion was carried out at 55°C for 2 hours. Organic extractions with a half volume of Phenol/Chloroform/Isoamyl alcohol (1:1:0.04) were repeatedly carried out until no visible interphase remained after centrifugation. DNA was precipitated from the aqueous phase in the presence of 0.3 M NaOAc pH 7.0 and two and a half volumes of ethanol and followed by one 70% ethanol-washing and dissolved at 65°C for 30 minutes with 0.2 – 0.4 ml TE (10 mM Tris-HCl pH 7.4 and 1 mM EDTA)and stored at 4°C till use. The DNA concentrations were calculated according to the OD_260 nm _readings.

#### Bisulphate treatment of DNA and Methylation specific PCR (MSP)

The methylation status of the promoter CpG islands of thirty four genes in all DNA samples was analyzed by MSP on the sodium-bisulfite converted DNA [18]. In detail, 10 μg DNA in 50 μl TE was incubated with 5.5 μl of 3 M NaOH at 37°C for 10 minutes, followed by a 16 hour treatment at 50°C after adding 30 μl of freshly prepared 10 mM hydroquinone and 520 μl of freshly prepared 3.6 M sodium-bisulfite at pH 5.0. The DNA was desalted using a home-made dialysis system with 1% agarose (detailed protocol will be provided upon request). The DNA in the desalted sample (approximately 100 μl in volume) was denatured at 37°C for 15 minutes with 5.5 μl of 3 M NaOH followed by ethanol precipitation with 33 μl 8 M NH_4_OAC and 300 μl ethanol. After washing with 70% ethanol, the gently dried DNA pellet was dissolved with 30 μl TE at 65°C for 10 min. The DNA sample was finally stored at -20°C until further use. PCR reaction was carried out in a volume of 15 μl with 50 ng or less template DNA with FastStart Taq polymerase (Roche, Germany) as follows. After an initial heat denaturing step 4 minutes treatment at 94°C, 30 cycles of 92°C for 15 sec, varying temperatures with primer pairs (Additional file 1) for 15 sec and 72°C for 20 sec, was carried out. The PCR products were separated by 1.2% ethidium bromide containing agarose gel electrophoresis with 1 × TAE and visualized under UV illumination. To verify the PCR results, representative bands from each target were gel-purified and cloned into T-vector (Promega, USA) followed by automatic DNA sequencing provided by BuoCai (Shanghai, China). Only verified results are presented in this report.

To optimize the MSP procedure, the M. Sss I treated DNA was used as the methylated control template. In detail, the DNA from a normal liver tissue of the healthy liver donor [18,24] was batch cleaved with EcoR I, followed by M. Sss I treatment according to the manufacture's instruction (New England Biol., Boston, USA) for over night. The purified DNA was bisulphate treated as usual and subjected to MSP with the primer pairs for each of thirty three genes (except for the *MAGEA1 *gene), and only the verified targets were included for the study of the astrocytoma tissues.

#### Statistical analysis

The methylation data were dichotomized as 1 for the co-existence of the methylated and unmethylated alleles, 2 for methylated allele only and 0 for the unmethylated for both alleles to facilitate statistical analysis using contingency tables. The methylation profiles of each individual gene (in percentage) classified by the genders and grading of the patients were presented both in table and in plot. The statistic analyses for the association between the methylation profile of the gene and each of the clinical-pathological parameters were carried out with the statistics package , where both Pearsong's Chi-square test with Upton's adjustment and Fisher exact test  were used to examine the tissue samples with the low expected values. The relative frequency with a 95% confidence interval (P < 0.05) for a binomial distribution was calculated for the whole as well as each subtype of astrocytoma patients.

The concordant methylation behavior of the genes was established by comparing frequency of co-occurrence of 2 to 3 target subsets with a mathematic method, namely Discovery of Association Rules [28], which is frequently utilized for association analysis.

#### Demethylation of U251 cells with 5-Aza-2'-deoxycytidine

U251 cells (an established glioma cell line) were cultured in DEME plus 10% new born calf serum at 37°C in a 5% CO_2 _atmosphere. When cell culture reached 50% confluence, they were treated with 5-Aza-2'-deoxycytidine (Sigma A3656) at the final concentration 10 and 20 nM, respectively for 3 days. The primer pairs for the RT-PCR (Table 1) was either adopted from published papers or designed with an assistance of the software . The total RNA was extracted with Trizol solution according to manufacturer's instruction (Invitrogen, USA), and cDNA was obtained using the Supertranscript plus reverse transcriptase with the oligo-dT as primers. PCR with single pair of the target primers run for 15 cycles, followed by another 15 cycle PCR reactions in the presence of the beta-actin primers (Table 1) (the parameter of each cycle is 94°C 20", 60°C for 20" and 72°C for 30"). The resulted PCR products were visualized under UV illumination after an electrophoretic separation on a 1.2% agarose. The methylation status of the target was analyzed by MSP.

**Table 1:** The primers for RT-PCR analysis

Primer Name	sequence	PCR Product Length (bp)	Accession Number
beta-actin L	AAGTACTCCGTGTGGATCGG	616	NM_001101
beta-actin R	TCAAGTTGGGGGACAAAAAG		
cdh13f	GCTGGACTGGATGTTGGATT	246	NM_001257
cdh13t	TTGAGGGTTGGTGTGGATTT		
magea1rf	ACCTGACCCAGGCTCTGT	401	NM_004988
magea1rt	CTCACTGGGTTGCCTCTG		
mgmtrf	AAACGCACCACACTGGAC	404	NM_002412
imgmtrt	AGGATGGGGACAGGATTG		
p73f	AGATGAGCAGCAGCCACAG	218	NM_005427
p73t	GTACTGCTCGGGGATCTTCA		
rassf1arf	GTCTGCCTGGACTGTTGC	401	NM_007182
rassf1art	AGCAGGGCCTCAATGACT		

## Results and discussion

### Clinical-pathological classification

To establish the methylation profile of thirty four genes during the process of astrocytoma development, we recruited 53 astrocytoma patients (27 female and 26 male; 49 primary and 4 recurrent) for this study. 14 cases were pathologically classified at the Grade I pilocytic astrocytoma (10–62 years old, mean: 39.1; 9 female, 5 male), 15 cases at the Grade II diffuse astrocytoma (4–50 years old, mean: 33.1; 10 female, 5 male), 12 cases at the Grade III anaplastic astrocytoma (1–72 years old; mean: 40.4; 4 female, 8 male), and 12 cases (including 4 recurrent cases) at the Grade IV glioblastoma (22–66 years old, mean: 44.6; SD = 22–66, 4 female, 8 male) (Table 2). The normal brain tissues distant from the lesions were also obtained from three non-astrocytoma patients who underwent brain surgery as normal controls in this study.

**Table 2:** The clinical and pathological profiles of the patients

			Astrocytoma	Non astrocytoma
Gender				
	female		27	1
	male		26	2
Age, y				
	<40		27	1
	40–60		23	2
	>60		3	0
Grade	Age			
			
	Mean	Range		
I	39.1	10 to 62	14	
II	33.1	4 to 50	15	
III	40.4	1 to 72	12	
IV	44.6	22 to 66	12	
	Recurrent		4	
	Primary		8	

### Aberrant Methylation profiling in astrocytoma

#### The technical considerations

The methylation-specific PCR (MSP) is widely used for methylation profiling of the genes in human cancers for both its easiness and sensitivity. However, the necessary steps have to be taken to eliminate both false positive and negative results. Comparing the MSP-data with the non-PCR data by Southern analysis of the methylation sensitive restriction enzyme is a valuable choice, as our previous work where the hypomethylated status of both *p14*^*ARF*^* and p15*^*INK4b *^genes shown by MSP was confirmed by Southern analysis [18]. Alternatively, the PCR reaction with the *in vitro *methylated genomic DNA (by M. Sss I) as template would be an ideal positive control for the absence of methylated targets in tumor tissue samples. By taking extra caution, we carried out MSP of all the targets with the M. Sss I treated normal liver DNA as positive control templates, except for the *MAGEA1 *gene was unmethylated in the normal liver tissue. While only the PCR reaction designated to the unmethylated template gave rise to the detectable bands with the parental DNA, the PCR bands were evident in both reactions with the M. Sss I treated DNA (Additional File 2). Therefore, failure to detect the methylated alleles with the tissue samples should genuinely reflect the lack of methylated targets. To control the false positive with either pair of primers, the representative PCR products, were T-cloned and sequenced. Only the positive PCR results with the expected sequence profiles were scored and analyzed further.

#### The methylation profiling of thirty four targets in astrocytoma

Eleven of the thirty four target genes were previously studied either in astrocytoma or other types of tumors. The published PCR conditions for these genes: *APC, BRCA1, CDH1, DAPK1, hMLH1, p14^ARF^, p15^INK4b^, p16^INK4a ^RASSF1A, RB1 *and *VHL *(Additional file 1) were adopted to enable the relevant inter-study comparisons if necessary. The remaining twenty three targets were selected from a list of genes  displaying the altered pattern of the promoter CpG island in various biological settings including cancers. Their CpG islands were identified via bioinformatical tools  and the primer pairs were designed accordingly [18,24]. Some of these thirty four genes have been shown to play a role in carcinogenesis, whereas the others have no obvious association with human carcinogenesis. Since it is still disputed whether DNA methylation mediated the gene silencing is causative in the malignant transformation of cell, we specifically selected both sets of genes in this study. The "cancer unrelated" genes selected encode erythropoiesis (*EPO*) [29], a ubiquitously expressed transcription factor (*OCT6*) [30], and the myogenesis lineage-specific transcription factor (*MYOD1*) [31]. The majority of the cancer associated genes examined were tumor suppressor genes including genes operating in the *RB1/p16*^*INK4a *^pathway (*p14^ARF^, p15^INK4b^, p16^INK4a^*, and *RB1*) [32], and two cyclin-dependent kinase inhibitors (*p27*^*KIP1 *^[33] and *p57*^*KIP2*^) [34]. Other genes in this subset were a *p53 *analogue:(*p73*) [33,35], two alternative forms of a tumor suppressors in the Ras mediated signal transduction pathway (*RASSF1A*, and *RASSF1C *[36]), *VHL *[37], *APC *[38], *PTEN *[6], the deleted in bladder cancer chromosome region candidate 1 (*DBCCR1*) [39], and the Wilms tumor 1 gene(*WT1*) [40]. We included the genes encoding the cell membrane proteins or nuclear receptors which act actively in the intercellular interactions: melanoma specific antigen A1 (*MAGEA1*) [41], caveolin 1 (*CAV*) [42], chondroitin sulfate proteoglycan 2 (*CSPG2*) [43], androgen receptor (*AR*) [44], and cadherins (*CDH1 *[45] and *CDH13*) [46]. Three genes implicated in signal transduction were also selected: cyclin a1 [47], the interferon regulatory factor 7 (*IRF7*), and a serine/threonine kinase 1 (Peutz-Jeghers syndrome) gene (*LKB1*) [14]. There were the genes encoding the O-6-methylguanine-DNA methyltransferase (*MGMT*) [14]and metallothionein 1 A gene (*MT1A*) [48] which play a key role in the cellular response to alkalyting agents and heavy metal stress. The genes acting in DNA repair process were *hMLH1 *[49], and *BRCA1 *[50], while four genes are involved in apoptosis (*APAF1 *[51], *DAPK1 *[15], and *SURVIVIN *[23]). Finally, the proto-oncogenes in this group were represented by v-abl homologue 1 (*ABL*) [52] (Additional files 3,4,5,6,7,8,9).

#### The genes displayed the uniformly unmethylated profiles in astrocytoma

Of the unmethylated genes in all samples tested, EPO was a cancer unrelated gene, while "cancer associated" genes included *ABL*(1), *APAF1*(2), *APC*(3), *BRCA1*(5), *CAV*(6), *CDH13*(8), *DAPK1*(11), *hMLH1*(14), *LKB1*(16), *p14*^*ARF*^(22), *p15*^*INK4b*^(23), *p27*^*KIP1*^(25), *p57*^*KIP2*^(26), *PTEN *(28), *RASSF1C*(30), *RB1*(31), *SURVIVIN*(32), and *VHL*(33) genes (Additional files 3,4,5,6,7,8,9).

Lack of hypermethylation of the RB1 gene in our observation was inconsistent with a recent report that the hypermethylated RB1 gene was detected in 19% of astrocytoma patients (26/136 cases analyzed) [53]. Since the same region was looked at in this work, the discrepancy noticed may simply reflect the inherent difference in the patient cohorts between our work and the published [53].

The genetic defects affecting the *PTEN *gene contributed to the pathogenesis of astrocytoma [54]. Lack of the hypermethylation of its promoter CpG island in both normal and astrocytoma tissues indicates that the DNA hypermethylation mediated silencing mechanism unlikely plays a significant role in the *PTEN *inactivation that occurs frequently in astrocytoma. This explanation might also be applicable to the no change type of methylation behavior for both the tumor associated genes (*ABL*(1), *APAF1*(2), *APC*(3), *BRCA1*(5), CAV(6), CDH13 (8), DAPK1(11), hMLH1(14), LKB1(16), p14^ARF^(22), p15^INK4b^(23), *p27*^*KIP1*^(25), *p57*^*KIP2*^(26), *PTEN *(28), *RASSF1C*(30), *RB1*(31), *SURVIVIN*(32), and *VHL*(33) genes) and the "cancer unrelated" genes (*EPO *(14)) (Additional files 3,4,5,6,7,8,9).

#### The genes with the astrocytoma specific alteration in methylation

**Figure 1:**
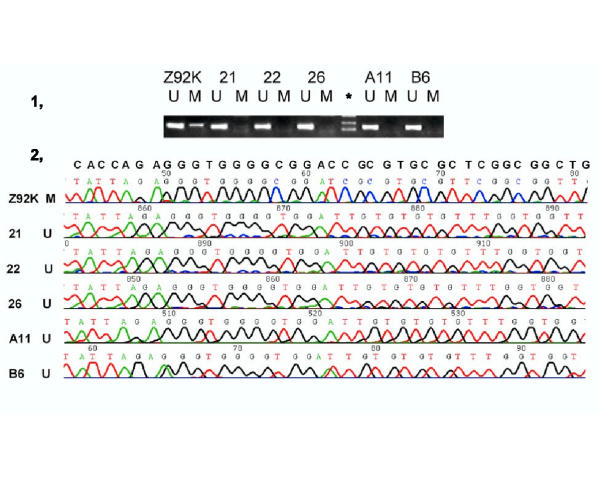
MSP/sequencing analyses of the p16^INK4a ^gene in astrocytoma and hepatocellular carcinoma Both electrophoretic patterns of the PCR products of the p16^INK4a ^in each of five astrocytoma cases (21, 22, 26, A11 and B6) and one HCC case (Z92K) (indicated respectively, at the top of figures) were presented. To indicate the methylation status, the sequenced data are aligned with the wild-type sequence.

As shown in Additional files 3,4,5,6,7,8,9, thirteen genes (*CDH1 *(7), *CSPG2*(9), *cyclin a1*(10), *DBCCR1*(12), *IRF7*(15), *MGMT*(18), *MT1A*(19), *MYOD1*(20), *OCT6*(21), *p16*^*INK4a *^(24), *p73*(27), *RASSF1A *(39) and *WT1*(34)) were unmethylated in all three normal controls. In contrast, these genes were hypermethylated to various extents in the astrocytoma samples. The following six genes were marginally hypermethylated: *p16*^*INK4a*^, *EPO*, *DBCCR1 *and *MYOD1 *genes were hypermethylated in 1.9% (1/53) of astrocytoma tissues, while both *CDH13 *and *cyclin a1 *genes were hypermethylated in 5.7% (3/53) of astrocytoma cases. No significant changes of these six genes shown in here acted against the notion that DNA methylation related mechanisms underline potential inactivation of this set of genes in the pathogenesis of astrocytoma. The infrequent hypermethylation of the *p16*^*INK4a *^gene in astrocytoma was a total surprise, as it was frequently reported hypermethylated in various human tumors tested, including in HCC where we have previously found that the *p16*^*INK4a*^, *MYOD1*, *CDH13 *and *cyclin a1 *genes were frequently methylated [18,24]. To further verify this unexpected observation, we repeated the MSP analysis on five astrocytoma samples (shown unmethylated) along with one HCC sample (previously shown heterozygously methylated). As shown in panel 1, Fig. 1, MSP patterns of the astrocytoma as well as HCC tissues remained the same. The identities of which were also confirmed by sequencing (panel 2, Fig. 1), showing that while the MSP products with the primers specific to the methylated targets in the HCC sample (Z92K) contained CpGs, the unmethylated targets in all the five astrocytoma tissues (21, 22, 26, A11 and B6) contained TpGs. Therefore, lack of hypermethylation of the *p16*^*INK4a *^gene in astrocytoma was unlikely incorrect, which is consistent with a recent report that inactivation of the *p16*^*INK4a *^gene in 48% of astrocytoma cases was genetic [55].

**Figure 2:**
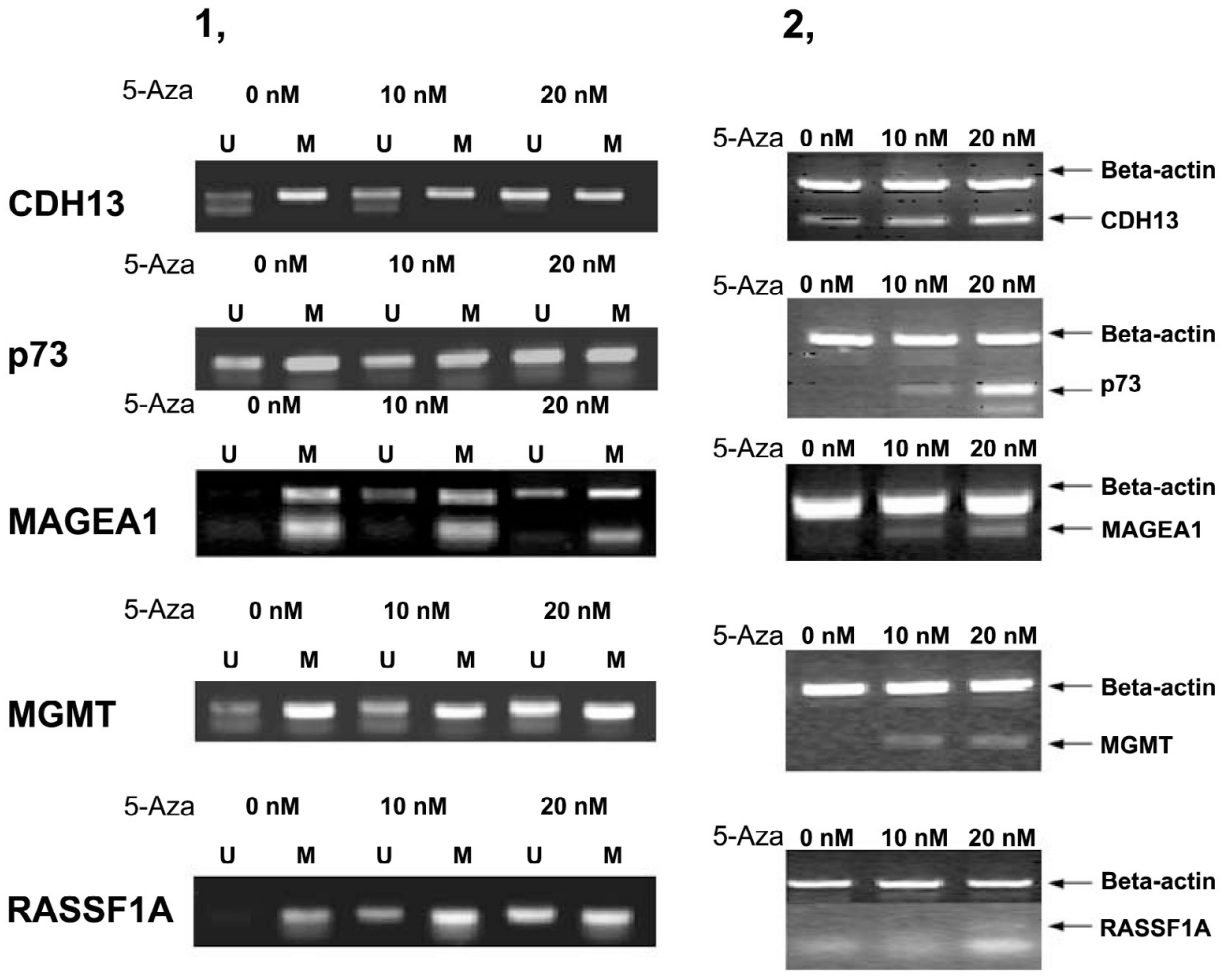
The methylation state and expression profiles of the CDH13, p73, MAGEA1, MGMT and RASSF1A genes in U251 astrocytoma cells before and after the demethylation treatment with 5-Aza-2'-deoxycytidine U251 cells were subjected to the 10 and 20 nM 5-Aza-2'-deoxycytidine (5-Aza) treatment for 3 days before both DNA and RNA were prepared for either MSP analyses or RT-PCR assessments. Panels; A, the methylation status of the CDH13, p73, MAGEA1, MGMT and RASSF1A genes and B, the expression profiles of each of these five genes, respectively in U251 cells.

The remaining 7 targets were hypermethylated more frequently, occurring in 26.4% to 69.8% (14 to 37/53) of astrocytoma cases. The *OCT6 *gene was hypermethylated in 30.2% of the astrocytoma cases (16/53). Despite of the association of the *OCT6 *methylation with the aging process reported previously, we found no significant correlation/association of the *OCT6 *methylation to any clinical-pathological features, including age, gender and clinical grading of the patients. The significance of such a prevalent occurrence of the hypermethylated *OCT6 *gene remains to be determined. The *RASSF1A *(hypermethylated in 37/53 cases, 69.8%) is a variant of the recently identified tumor suppressor, the *RASSF1 *gene that acts at downstream of the Ras mediated apoptotic pathway and is capable of binding to Ras in a GTP dependent manner [36]. The *RASSF1A *gene has a more extended 5' part and its promoter CpG island displays a tumor specific hypermethylated profile in a variety of tumors, HCC in particular. Furthermore, lack of the RASSF1A expression in nineteen established tumor cell lines correlates with the hypermethylated state of its promoter CpG island [36]. The *RASSF1C *gene has its own promoter CpG island, but is not methylated in any tumors. The methylation behavior of these two genes was very similar to our previous observation in hepatocellular carcinoma, where 22/29 cases (79%) had the fully methylated 1A along with the unmethylated 1C variants [18]. As shown in Additional file 4,5,6,7,8,9, the *RASSF1A *promoter-CpG island was methylated in 69.8% (37/53) of astrocytoma tissues, while the C variant was not methylated in any astrocytoma tissues. The hypermethylated state of the *RASSF1A *promoter CpG island was not correlated with gender, age and clinical grading. Consistent with the hypermethylated status of the *RASSF1A *gene in U251 cells, no expression at the mRNA level was detected. Partial demethylation of its promoter by the treatment with 5-Aza-2'-deoxycytidine indeed resulted in its transcription (Fig. 2).

The *p73 *gene encodes a homologue to TP53, and loss of its heterozygosity has been observed in up to 90% of oligodendrogliomas and in 10–25% of diffuse astrocytoma [56,57]. In this study, we found that the *p73 *gene was prevalently methylated (25/53, 47.2%) with no significant association with any clinical-pathological parameters, such as gender and the WHO grading. The occurrence of the hypermethylated *p73 *gene was more prevalent in our results than a recent report which detected the hypermethylated *p73 *gene in 18% (5 /28) of the WHO grade IV but not in grade III astrocytoma [35]. Again, even the partially elevated demethylated status of its promoter CpG island in U251 cells resulted in reactivation of *p73 *transcription (Fig. 2).

Both genetic defects and epigenetic abnormalities of the *WT1 *gene have been etiologically implicated in the formation of the Wilm's tumor [58]. In this study, we also found that the *WT1 *gene was hypermethylated in 30% (16/53) of cases, implying its possible involvement in the formation of astrocytoma.

Tumor resistance to the cytotoxic chemotherapies may result from the disrupted apoptosis programs and remains a major obstacle in cancer treatment. In this study, the interferon regulatory factor 7 (*IRF7*) gene was analyzed. The analogue (*IRF1*) of IRF7 has been implicated in the IFN gamma mediated apoptosis with a profound effect on the chemo-sensitivity of tumor cells [59,60]. In consistence with the recent report that the IRF7 expression was negatively regulated by the promoter methylation [61], we found that the IRF7 gene was hypermethylated in astrocytoma (14/53, 26.5%) (Additional file 4,5,6,7,8,9), with a strong male inclination (11/26, 42.3% verse the female group: 3/27, 11%, χ^2 ^= 6.632, P = 0.014). Although the gender difference remains to be understood, such a strong male association with IRF7 hypermethylation may have prognostic value.

O(6)-methylguanine-DNA methyltransferase (MGMT), a DNA repair enzyme, removes alkylating adducts from the O(6) position of guanine and protects cells from cytotoxic and mutagenic stress. Silencing of the *MGMT *gene has been suggested to predispose the neoplastic clones to acquisition of the guanine to adenine point mutations in K-*ras *and *p53 *[62] and is associated with low-levels of micro-satellite instability in colorectal cancer [63]. We found that the *MGMT *gene was prevalently hypermethylated in astrocytoma (35%, 19/53), and its transcription could be reactivated by demethylation with 5-Aza-2'-deoxycytidine in U251 cells (Fig. 2). Hence, the *MGMT *hypermethylation in astrocytoma may indeed have the pathological significance. In this connection, a recent report suggested that the astrocytoma sensitivity to the alkylating type of chemotherapeutics might be contributed by the hypermethylated *MGMT *gene [64]. Expression of the metallothionein I A (*MT1A*) is inducible by a number of adversary agents such as heavy metals and oxidative stress. Both basal and inducible expression of this gene has been impaired in various tumor cell lines and attributed to the hypermethylated state of this gene [48]. In this study, we found that the *MT1A *gene was hypermethylated in 30% (16/53) of cases, with no significant gender and grading difference. The functional and pathological implications of the *MT1A *hypermethylation in astrocytoma remain to be established.

Cadherins, the calcium-dependent proteins, contribute to various biological processes such as differentiation, migration and extra-cellular signal transduction of cell. Loss of expression of both E-cadherin (*CDH1*) and H-cadherin (*CDH13*) has been found in parallel with the hypermethylated promoter CpG islands in various cancers [65,66]. In this study, we found that the *CDH1 *gene was hypermethylated in 32.8% (17/53) of astrocytoma tissues, while the *CDH13 *gene was not methylated in all the astrocytoma tissues examined (Additional files 4,5,6,7,8,9). In contrast, in human hepatocellular carcinoma [18], the *CDH1 *gene was unmethylated, while the CDH13 *gene *was frequently hypermethylated. Obviously, the molecular basis for tumor type specific methylation patterns of these two genes remains to be determined.

Although the hypermethylation mediated gene silencing of the tumor suppressor genes is at the focal point of the epigenetic studies, demethylated status of the promoter CpG islands has been linked to the tumor associated activation of the normally silenced genes [19-23]. Therefore, we also studied both *MAGEA1 *and *SURVIVIN *genes. The promoter CpG islands were hypermethylated in normal tissues (for *MAGEA1 *in HCC [18] and for *SURVIVIN *in ovarian cancer [23]) and demethylated in parallel with the transcriptional activation in tumor cells. The unmethylated status of the *SURVIVIN *gene in astrocytoma is consistent with the over-expression of this gene (unpublished observations). However, its unmethylated status in all the non-astrocytoma tissues acts odd with the notion that its demethylation is associated with pathogenesis in human ovarian cancer reported previously [23].

Our previous studies indicated that demethylation of the promoter CpG island was correlated well with the over-expression profile of the *MAGEA1 *gene [18,21] in HCC. The *MAGEA1 *gene was fully hypermethylated in all four cases of the normal liver tissues but significantly demethylated in HCC tissues (21/28, 75%). It was found fully hypermethylated in all the three control tissues and in 74.5% (40/53) of the astrocytoma tissues and partially hypermethylated (13/53, 25.5%) in the other astrocytoma tissues. The occurrence of the *MAGEA1 *demethylation in HCC differed significantly from astrocytoma (75% verse 25.5%, P < 0.001). As it was fully methylated in the normal tissue, the partial hypermethylation (both hypermethylated and demethylated alleles existed) would imply that the event resulting in the loss of the hypermethylation state of the *MAGEA1 *gene indeed occurred in astrocytoma and should be scored positive for the changes in the methylation pattern in this study. The same principle has been applied for the opposite changes from the unmethylated pattern in the normal control to the partial or full hypermethylated status of all the other genes in astrocytoma tissues. It was also found that the partial demethylated status of the *MAGEA1 *gene in U251 cells induced by 5-Aza-2'-deoxycytidine occurred co-currently with activation of its transcription (Fig. 2).

#### The gender association of the methylation profiles of the AR and IRF7 gene in astrocytoma

By statistic analysis with both Pearson Chi-Square and Fisher's Exact tests, associations of the DNA methylation profiles of the targets displaying no less than 24.5% changes (the *RASSF1A, p73, MGMT, CDH1, OCT6, WT1 *as well as *MAGEA1 *genes) with the clinical pathological parameters (age, grading and gender) were assessed. The methylation profiles of the *AR *and *IRF7 *genes were found gender-oriented.

The *AR *gene encodes the androgen receptor that plays a key role in the signal transduction pathways in response to the male steroid hormone, androgen and has been reported to be inactivated via the epigenetic mechanism in prostate cancers [67]. Physiologically, the AR gene should express exclusively in the somatic cells in males, while lacking of its expression in females is likely mediated by DNA methylation based mechanisms. Indeed, the hypermethylated along with the unmethylated AR genes were only found in the normal female brain tissue, but not from two male non-astrocytoma samples. The hypermethylation of the AR gene occurred frequently in the female group (81.5%, 22/27) but not in any males (0%, 0/26, χ^2 ^= 36.22, P = 0.000). It may simply be gender associated and do not have any significant relevance to carcinogenesis of astrocytoma. It was also noticed that hypermethylation of the IRF7 gene displayed an opposite gender inclination, detected in 11% of the female patients (3/27), and 42% of male patients (11/26, χ^2 ^= ?6.632, P = 0.014). Despite of the difficulty to offer a mechanistic interpretation, the potential prognostic value of such a gender-associated phenomenon might be worthwhile exploring in future.

#### Demethylation by 5-Aza-2'-deoxycytidine treatment of the astrocytoma cells in culture resulted in partial demethylation and reactivated expression of the genes

The hypermethylated status of the promoter CpG island has been linked to gene transcription silencing in a number of biological settings. The effect of the astrocytoma associated changes in the methylated state of the promoter CpG islands detected in this study on gene expression was assessed in U251 astrocytoma cells treated with the a demethylating agent, 5-Aza-2'-deoxycytidine. We used MSP to establish the methylation status of the promoter CpG island of all the genes with the astrocytoma associated methylation changes (Additional files 3,4,5,6,7,8,9) in U251 astrocytoma cells, and analyzed the ability of 5-Aza-2'-deoxycytidine to demethylate five genes, as measured by MSP, and reactivate their expression, as detected by RT-PCR.

As shown in panel 1 of Fig. 2, while the *CDH13*, *MAGEA1 *and *p73 *genes were heterozygously methylated, both *MGMT *and *RASSF1A *genes were fully hypermethylated in U251 cells. The *CDH13 *gene was found expressed, while the rest transcriptionally inert as measured by the RT-PCR. Although both methylated and unmethylated alleles for *p73 *and *MGMT *genes were evident in U251 cells, no expression was detected, indicating that the unmethylated allele may remain silent by the other mechanisms, including the genetic defects at critical control region. By the 5-Aza-2'-deoxycytidine treatment, both demethylation of the promoter CpG island and activation of transcription of these five genes were achieved (Fig. 2). Despite of the fact that demethylation of the promoter CpG islands was incomplete in samples treated with 20 nM 5-Aza-2'-deoxycytidine (Fig. 2), the expression of this five genes was either induced (the *MAGEA1*, *MGMT*, *p73 *and *RASSF1A *genes) or elevated (the *CDH13 *gene).

#### The concordant methylation behavior of the promoter CpG islands of the genes in Astrocytoma

The DNA methylation mediated epigenetic changes also display the tumor type specific patterns, which seem to reflect the differentiation and maturation histories of the cell lineages as well as the aging process during which both global hypo- and local hyper-methylation occur. Hypermethylation of the promoter CpG islands in accord with the transcriptional silencing of the tumor suppressor genes, such as the p16^INK4a^, and RASSF1A genes, has been well established in human tumors [16,68]. However, it remains unclear whether there is a common mechanism for the concurrent methylation changes of multiple tumor suppressor genes in tumors. To address this matter, it is necessary to examine a large number of genes for frequent changes in methylation in any type of human tumors. The concordant methylation behavior of multiple genes was firstly detected in colon cancer [69], based upon a comprehensive methylation profiling of over thirty genes. In this study, we have profiled the methylation status of thirty four genes in a cohort of 53 astrocytoma and 3 non-astrocytoma patients. Twenty three of these genes had not been studied previously in astrocytoma. As far as the number of the genes is concerned, this study is the most extensive in the astrocytoma field to our knowledge. Among thirty four genes, sixteen genes exhibited the astrocytoma associated changes in methylation profiles of the promoter CpG islands and nine genes displayed rather frequent changes (the occurrence ≥ 13/53, frequency ≥ 24.5%) (Additional file 8).

Four of 53 cases (7.5%) maintained the same methylation profile as the normal control. The rest 49 cases (92.5%) suffered from the methylation changes as much as no less than one target, an occurrence was significantly lower than in HCC, where all the cases displayed methylation changes affecting no less than three targets in the studies involved with twenty or twenty four targets [18,24], indicating that alterations in DNA methylation \occur more frequently in HCC than in astrocytoma. This may be contributed by the apparent anatomic inaccessibility of the brain to environmental adverse factors in comparison to the liver. The size of the subsets containing various number of the target affected (from one to nine) ranged from 1 to 11 cases, and peaked with 10 cases at three and 11 cases at five target subsets (Additional file 9). To identify the most frequent changes of the target sets (one to three), a mathematic method called "the Discovery of Association Rules" [28] was used. The co-occurrence (case number/the total) and frequency (% of the total) of any subset of the targets that changed in methylation together in astrocytoma were counted and compared. In the entire cohort of patients in this study, the most altered target was the RASSF1A gene, 69.8% (37/53). The two genes that most altered together were the *RASSF1A *and *p73 *genes, hypermethylation of which was found in 20 (37.7%). Three genes that changed together were the former two plus *CDH1 *or *OCT6*, hypermethylation of which occurred in 20.8% cases (11/53) (Column 2, a, Additional file 10). Furthermore, the occurrence in methylation change in any target in the two gene subsets was 79.3% (42/530 and in three gene subsets was 81.1–83% (43–44/53) (Column 3, a, Additional file 10).

Since the hypermethylated *AR *is associated closely with the female gender of the astrocytoma patients and devoid of any association with the formation of astrocytoma, it was taken out from this analysis. Hypermethylation of the *RASSF1A *gene occurred in 21 female cases (77.8%, 21/27). Both *RASSF1A *and *WT1 *were hypermethylated in 13 (13/27, 48.1%); and the former two plus the hypermethylated *p73 *or *CDH1 *or *OCT6 *were found in 9 female cases (9/27, 33.3%), respectively (Column 2, b, Additional file 10). The subsets in the male patient group showed very different patterns. The single to three target subsets were the *RASSF1A *(16/26, 61.5%); the *RASSF1A *and *IRF7 *(10/26, 38.5%); and the former two plus the *p73 *or *MGMT *or *MT1A *(5/26, 19.2%), respectively (c, Additional file 10). In Grade I astrocytoma, the subsets for one, two and three targets were *RASSF1A *(10/14, 71.4%), *RASSF1A *plus p73 (6/14, 42.9%), and the former two plus either *WT1 *or *IRF7 *or *MAGEA1 *as well as *RASSF1A *plus *CDH1 *and *WT1 *(3/14, 21.4%). For Grade II astrocytoma, the corresponding sets consisted of the *RASSF1A *(12/15, 80%), the *RASSF1A *and *MGMT *or *IRF7 *(5/15, 33.3%), and the *RASSF1A *and *MGMT *plus *p73 *or *OCT6*, or *MT1A*, or *WT1 *as well as the *RASSF1A *and *IRF7 *and *MT1A *(3/15, 20%), respectively. For Grading III astrocytoma, those subsets were composed of the *RASSF1A *(8/12, 66.7%), the *RASSF1A *and *CDH1 *(5/12, 41.7%), and the formal two plus *MGMT *(4/12, 33.3%), respectively. For Grading IV astrocytoma, the comparative subsets contained the *RASSF1A *or *p73 *(7/12, 58.3%), the *RASSF1A *and *p73 *(6/12, 50%), and the former two plus *MGMT *or *OCT6 *(4/12, 33.3%), respectively. (d-g, Additional file 10).

Our methylation profiling efforts described in this report provided the following informative targets: the *RASSF1A*, *p73*, *WT1*, *MGMT*, *CDH1*, *OCT6*, and *IRF7 *genes. The established concordant methylation profiles of these eight genes (Additional file 10) may provide useful clues for the epigenetic biomarker selection to for the novel diagnostic and prognostic assays of astrocytoma. The hypermethylated status of this lest of genes in the serum, and biopsies of the suspected astrocytoma patients may serve as good diagnostic indicators, if they can be reliably detected. With the death/survival profiles of this cohort of astrocytoma patients available in the future, the methylation profile established in this study may have certain prognostic value.

## Abbreviations

HCC: Hepatocellular carcinoma; PCR: polymerase chain reaction; MSP: methylation specific PCR; *ABL*: v-abl Abelson murine leukemia viral oncogene homolog 1; *APAF1*: apoptotic protease activating factor; *APC*: adenomatosis polyposis coli; *AR*: androgen receptor; *BRCA1*: breast cancer 1; *CAV*: caveolin 1, caveolae protein; *CDH1*: cadherin type 1, E-cadherin; *CDH13*: cadherin 13, H-cadherin; *CSPG2*: chondroitin sulfate proteoglycan 2 (versican); *cyclin a1*: cyclin A1; *DAPK1*: death-associated protein kinase 1; *DBCCR1*: deleted in bladder cancer chromosome region candidate 1; *EPO*: erythropoietin; *hMLH1*: mutL homolog 1, colon cancer, nonpolyposis type 2; *IRF7*: interferon regulatory factor 7; *LKB1*: serine/threonine kinase 11 (Peutz-Jeghers syndrome); *MAGEA1*: melanoma antigen, family A, 1 (directs expression of antigen MZ2-E); *MGMT*: O-6-methylguanine-DNA methyltransferase; *MT1A*: metallothionein 1A (functional); *MYOD1*: myogenic factor 3; *OCT6*: POU domain, class 3, transcription factor 1; *p14*^*ARF*^: the alternative reading frame of the cyclin-dependent kinase inhibitor 4a; *p15*^*INK4b*^: cyclin-dependent kinase inhibitor 4b; *p16*^*INK4a*^: cyclin-dependent kinase inhibitor 4a; *p27*^*KIP1*^: cyclin-dependent kinase inhibitor 1B (p27, KIP1); *p57*^*KIP*2^: cyclin-dependent kinase inhibitor 1C (p57, KIP2); *p73*: tumor protein p73; *PTEN*: phosphatase and tensin homolog; *RASSF1A*: ras association (RalGDS/AF-6) domain family 1 protein isoform 1a; *RASSF1C*: ras association (RalGDS/AF-6) domain family 1 protein isoform 1c; RB1: retinoblastoma 1; *VHL*: von Hippel-Lindau syndrome; *WT1*: Wilms tumor 1.

## Competing interests

None declared.

## Authors' contributions

JY, HYZ, JG, executing the experiments;

SL and JHL, providing the patient samples;

WL and YFW, carrying out the mathematic analyses of the data

JDZ: designing and organizing experiments as well as completing manuscript.

## Pre-publication history

The pre-publication history for this paper can be accessed here:



## Supplementary Material

Additional File 1 The target promoter CpG islands and the primer pairs for methylation specific PCR. This file contains his study. Click here for file

Additional File 2 Methylation profiles of thirty three genes on the *in vitro *methylated genomic DNA by M. Sss I methyl transferase. The Eco RI restricted genomic DNA from the liver tissue of a healthy donor was *in vitro *methylated overnight with M. Sss I methyl transferase and subjected to the MSP analysis, followed by electrophoresis in a 1.3% agarose gel. *, the DNA size markers, NL, the untreated sample, U and M, MSP with the pair of primers specific to the unmethylated and methylated targets, respectively. Panels: 1, ABL; 2, APAF1; 3, APC; 4, AR; 5, BRCA1; 6, CAV; 7, CDH1; 8, CDH13; 9, CSPG2; 10, cyclin a1; 11, DAPK1; 12, DBCCR1; 13, EPO; 14, hMLH1; 15, IRF7; 16, LKB1; 17, MGMT; 18, MT1A; 19, MYOD1; 20, OCT6; 21, p14^ARF^; 22, p15^INK4b^; 23, p16^INK4a^; 24, p27 ^KIP1^; 25, p57^KIP2^; 26, p73; 27, PTEN; 28, RASSF1A; 29, RASSF1C; 30, RB1; 31, SURVIVIN; 32, VHL and 33, WT1. Click here for file

Additional File 3 Methylation profiles of thirty four genes in astrocytoma (part I). Both electrophoretic patterns of the representative PCR products of each of thirty four targets (indicated respectively, at the top of figures) and the sequencing verification of the one representative PCR product were presented. To indicate the methylation status, the sequenced data are aligned with the wild-type sequence. *, size markers, the bands of 250 bp and 100 bp were shown. U, the unmethylated; M, the hypermethylated. Panels: 1, ABL; 2, APAF1; 3, APC; 4, AR; 5, BRCA1; 6, CAV; 7, CDH1; 8, CDH13; 9, CSPG2; 10, cyclin a1; 11, DAPK1 and 12, DBCCR1. Click here for file

Additional File 4 Methylation profiles of the promoter CpG islands of thirty four genes in astrocytoma (part II). Both electrophoretic patterns of the representative PCR products of each of thirty four targets (indicated respectively, at the top of figures) and the sequencing verification of the one representative PCR product were presented. To indicate the methylation status, the sequenced data are aligned with the wild-type sequence. *, size markers, the bands of 250 bp and 100 bp were shown. U, the unmethylated; M, the hypermethylated. Panels: 13, EPO; 14, hMLH1; 15, IRF7; 16, LKB1; 17, MAGEA1; 18, MGMT; 19, MT1A; 20, MYOD1; 21, OCT6 and 22, p14^ARF>^. Click here for file

Additional File 5 Methylation profiles of the promoter CpG islands of thirty four genes in astrocytoma (part III). Both electrophoretic patterns of the representative PCR products of each of thirty four targets (indicated respectively, at the top of figures) and the sequencing verification of the one representative PCR product were presented. To indicate the methylation status, the sequenced data are aligned with the wild-type sequence. *, size markers, the bands of 250 bp and 100 bp were shown. U, the unmethylated; M, the hypermethylated. Panels: 23, p15^INK4b^; 24, p16^INK4a^; 25, p27 ^KIP1^; 26, p57^KIP2^; 27, p73; 28, PTEN; 29, RASSF1A; 30, RASSF1C; 31, RB1; 32, SURVIVIN; 33, VHL and 34, WT1. Click here for file

Additional File 6 The summary of the astrocytoma cases displaying no or changes in the methylation profiles (part I). The frequency (%) of the astrocytoma displaying no or the changes in the methylation profile of each target from the normal control were counted and presented in table as well as plotted in the figure below. The filled, shading and empty boxes indicate the cases where only hypermethylated allele, both hypermethylated and unmethylated alleles and only unmethylated alleles were detected, respectively. The frequency (%) of the hypermethylated targets (except for the MAGEA1 gene) among the total cases was scored for positive changes in astrocytoma. The MAGEA1 was fully methylated (3/3, 100%) in the control, and become partially demethylated in some astrocytoma, therefore, demethylation of the MAGEA1 in astrocytoma was scored as positive changes. Sub-tables: a, the female patient group, b, the male patient group, and c, the control. Click here for file

Additional File 7 The summary of the astrocytoma cases displaying no or changes in the methylation profiles (part II). The frequency (%) of the astrocytoma displaying no or the changes in the methylation profile of each target from the normal control were counted and presented in table as well as plotted in the figure below. Sub-tables d-h, the WHO grading I to IV, respectively; The filled, shading and empty boxes indicate the cases where only hypermethylated allele, both hypermethylated and unmethylated alleles and only unmethylated alleles were detected, respectively. The frequency (%) of the hypermethylated targets (except for the MAGE**A**1, where the heterozygously hypermethylated) among the total cases was presented in the plot. Click here for file

Additional File 8 The occurrences and frequency of changes in methylation. *, One of three cases was methylated; **, The MAGEA1 gene was fully methylated in the normal tissues and partially demethylated in astrocytoma patients as indicated in the relevant cells. Therefore, the astrocytoma associated changes in methylation of this gene is opposite to the rest, i.e., demethylation rather than hypermethylation. Figure is each cells are the frequency in % and occurrence (case number). Click here for file

Additional File 9 The summary of changes in the methylation pattern in subsets. Both occurrence (case number) and frequency (%) for the subsets having no change in methylation and changes in one to nine genes are presented in % and (case number) in the top half of table, which was also plotted. Both occurrence (case number) and frequency (%) for the subsets having no change in methylation and changes in, at least, one to nine genes are presented in % and (case number) in the bottom half of table. Click here for file

Additional File 10 The summary of the concordant methylation behavior of the hypermethylated targets in astrocytoma. The co-occurrence (/total case) and frequency (%) of a panel subsets consisting of one to three targets were treated with method "Discovery Association Rules" and presented. Sub-tables: a, the total, b, the female, c, the male, and d-g, the grade I to IV, respectively. Column 1 is the number of target in each subset. Column 2 is the co-occurrence (case number/total) (frequency in %). Column 3 is the occurrence of any single target in each subsets, presented in case number (frequency %). The column 4 is the gene(s) in subset. N.B., In view of the strong female inclination of the AR methylation and lacking of any association with astrocytoma, AR has been taken off from this analyses. Click here for file

## References

[B1] Kleihues P, Cavenee W, Kleihues P, Cavenee W (2000). Astrocytic tumours. Pathology & Genetics Tumours of the Nervous System.

[B2] (1998). Cancer Incidence and Mortality in China, 1993–1997 (Selected Cities and Counties).

[B3] Libermann TA, Nusbaum HR, Razon N, Kris R, Lax I, Soreq H, Whittle N, Waterfield MD, Ullrich A, Schlessinger J (1985). Amplification, enhanced expression and possible rearrangement of EGF receptor gene in primary human brain tumours of glial origin. Nature.

[B4] Hegi ME, zur Hausen A, Ruedi D, Malin G, Kleihues P (1997). Hemizygous or homozygous deletion of the chromosomal region containing the p16INK4a gene is associated with amplification of the EGF receptor gene in glioblastomas. Int J Cancer.

[B5] Ichimura K, Bolin MB, Goike HM, Schmidt EE, Moshref A, Collins VP (2000). Deregulation of the p14ARF/MDM2/p53 pathway is a prerequisite for human astrocytic gliomas with G1-S transition control gene abnormalities. Cancer Res.

[B6] Ichimura K, Schmidt EE, Goike HM, Collins VP (1996). Human glioblastomas with no alterations of the CDKN2A (p16INK4A, MTS1) and CDK4 genes have frequent mutations of the retinoblastoma gene. Oncogene.

[B7] Mashiyama S, Murakami Y, Yoshimoto T, Sekiya T, Hayashi K (1991). Detection of p53 gene mutations in human brain tumors by single-strand conformation polymorphism analysis of polymerase chain reaction products. Oncogene.

[B8] Li J, Yen C, Liaw D, Podsypanina K, Bose S, Wang SI, Puc J, Miliaresis C, Rodgers L, McCombie R, Bigner SH, Giovanella BC, Ittmann M, Tycko B, Hibshoosh H, Wigler MH, Parsons R (1997). PTEN, a putative protein tyrosine phosphatase gene mutated in human brain, breast, and prostate cancer. Science.

[B9] Jones PA (2003). Epigenetics in carcinogenesis and cancer prevention. Ann N Y Acad Sci.

[B10] Feninberg A (2001). Cancer epigenetics takes center stage. Proc Natl Acad Sci U SA.

[B11] Eden A, Gaudet F, Waghmare A, Jaenisch R (2003). Chromosomal instability and tumors promoted by DNA hypomethylation. Science.

[B12] Gaudet F, Hodgson JG, Eden A, Jackson-Grusby L, Dausman J, Gray JW, Leonhardt H, Jaenisch R (2003). Induction of tumors in mice by genomic hypomethylation. Science.

[B13] Chen RZ, Pettersson U, Beard C, Jackson-Grusby L, Jaenisch R (1998). DNA hypomethylation leads to elevated mutation rates. Nature.

[B14] Esteller M, Fraga MF, Guo M, Garcia-Foncillas J, Hedenfalk I, Godwin AK, Trojan J, Vaurs-Barriere C, Bignon YJ, Ramus S, Benitez J, Caldes T, Akiyama Y, Yuasa Y, Launonen V, Canal MJ, Rodriguez R, Capella G, Peinado MA, Borg A, Aaltonen LA, Ponder BA, Baylin SB, Herman JG (2001). DNA methylation patterns in hereditary human cancers mimic sporadic tumorigenesis. Hum Mol Genet.

[B15] Zochbauer-Muller S, Fong KM, Virmani AK, Geradts J, Gazdar AF, Minna JD (2001). Aberrant promoter methylation of multiple genes in non-small cell lung cancers. Cancer Res.

[B16] Rosas SL, Koch W, da Costa Carvalho MG, Wu L, Califano J, Westra W, Jen J, Sidransky D (2001). Promoter hypermethylation patterns of p16, O6-methylguanine-DNA-methyltransferase, and death-associated protein kinase in tumors and saliva of head and neck cancer patients. Cancer Res.

[B17] Foster SA, Wong DJ, Barrett MT, Galloway DA (1998). Inactivation of p16 in human mammary epithelial cells by CpG island methylation. Mol Cell Biol.

[B18] Yu J, Ni M, Xu J, Zhang H, Gao B, Gu J, Chen J, Zhang L, Wu M, Zhen S (2002). Methylation profiling of twenty promoter-CpG islands of genes which may contribute to hepatocellular carcinogenesis. BMC Cancer.

[B19] Cui H, Onyango P, Brandenburg S, Wu Y, Hsieh CL, Feinberg AP (2002). Loss of imprinting in colorectal cancer linked to hypomethylation of H19 and IGF2. Cancer Res.

[B20] Cho B, Lee H, Jeong S, Bang YJ, Lee HJ, Hwang KS, Kim HY, Lee YS, Kang GH, Jeoung DI (2003). Promoter hypomethylation of a novel cancer/testis antigen gene CAGE is correlated with its aberrant expression and is seen in premalignant stage of gastric carcinoma. Biochem Biophys Res Commun.

[B21] Mou DC, Cai SL, Peng JR, Wang Y, Chen HS, Pang XW, Leng XS, Chen WF (2002). Evaluation of MAGE-1 and MAGE-3 as tumour-specific markers to detect blood dissemination of hepatocellular carcinoma cells. Br J Cancer.

[B22] Suyama T, Ohashi H, Nagai H, Hatano S, Asano H, Murate T, Saito H, Kinoshita T (2002). The MAGE-A1 gene expression is not determined solely by methylation status of the promoter region in hematological malignancies. Leuk Res.

[B23] Hattori M, Sakamoto H, Satoh K, Yamamoto T (2001). DNA demethylase is expressed in ovarian cancers and the expression correlates with demethylation of CpG sites in the promoter region of c-erbB-2 and survivin genes. Cancer Lett.

[B24] Yu J, Zhang HY, Ma ZZ, Lu W, Wang YF, Zhu JD (2003). Methylation profiling of twenty four genes and the concordant methylation behaviours of nineteen genes that may contribute to hepatocellular carcinogenesis. Cell Res.

[B25] Alonso ME, Bello MJ, Gonzalez-Gomez P, Arjona D, Lomas J, de Campos JM, Isla A, Sarasa JL, Rey JA (2003). Aberrant promoter methylation of multiple genes in oligodendrogliomas and ependymomas. Cancer Genet Cytogenet.

[B26] Gonzalez-Gomez P, Bello MJ, Arjona D, Lomas J, Alonso ME, De Campos JM, Vaquero J, Isla A, Gutierrez M, Rey JA (2003). Promoter hypermethylation of multiple genes in astrocytic gliomas. Int J Oncol.

[B27] Clark SJ, Harrison J, Paul CL, Frommer M (1994). High sensitivity mapping of methylated cytosines. Nucleic Acids Res.

[B28] Agrawal R, Imielinski T, Swami A (1993). Mining association rules between sets of items in large databases. ACM SIGMOD Conference.

[B29] Yin H, Blanchard KL (2000). DNA methylation represses the expression of the human erythropoietin gene by two different mechanisms. Blood.

[B30] Sauter P, Matthias P (1998). Coactivator OBF-1 makes selective contacts with both the POU-specific domain and the POU homeodomain and acts as a molecular clamp on DNA. Mol Cell Biol.

[B31] Chen B, Dias P, Jenkins JJ, Savell VH, Parham DM (1998). Methylation alterations of the MyoD1 upstream region are predictive of subclassification of human rhabdomyosarcomas. Am J Pathol.

[B32] Sherr CJ, McCormick F (2002). The RB and p53 pathways in cancer. Cancer Cell.

[B33] Kibel AS, Christopher M, Faith DA, Bova GS, Goodfellow PJ, Isaacs WB (2001). Methylation and mutational analysis of p27(kip1) in prostate carcinoma. Prostate.

[B34] Li Y, Nagai H, Ohno T, Yuge M, Hatano S, Ito E, Mori N, Saito H, Kinoshita T (2002). Aberrant DNA methylation of p57(KIP2) gene in the promoter region in lymphoid malignancies of B-cell phenotype. Blood.

[B35] Watanabe T, Huang H, Nakamura M, Wischhusen J, Weller M, Kleihues P, Ohgaki H (2002). Methylation of the p73 gene in gliomas. Acta Neuropathol (Berl).

[B36] Agathanggelou A, Honorio S, Macartney DP, Martinez A, Dallol A, Rader J, Fullwood P, Chauhan A, Walker R, Shaw JA, Hosoe S, Lerman MI, Minna JD, Maher ER, Latif F (2001). Methylation associated inactivation of RASSF1A from region 3p21.3 in lung, breast and ovarian tumours. Oncogene.

[B37] Linehan WM, Lerman MI, Zbar B (1995). Identification of the von Hippel-Lindau (VHL) gene. Its role in renal cancer. Jama.

[B38] Neibergs HL, Hein DW, Spratt JS (2002). Genetic profiling of colon cancer. J Surg Oncol.

[B39] Habuchi T, Luscombe M, Elder PA, Knowles MA (1998). Structure and methylation-based silencing of a gene (DBCCR1) within a candidate bladder cancer tumor suppressor region at 9q32-q33. Genomics.

[B40] Laux DE, Curran EM, Welshons WV, Lubahn DB, Huang TH (1999). Hypermethylation of the Wilms' tumor suppressor gene CpG island in human breast carcinomas. Breast Cancer Res Treat.

[B41] De Smet C, De Backer O, Faraoni I, Lurquin C, Brasseur F, Boon T (1996). The activation of human gene MAGE-1 in tumor cells is correlated with genome-wide demethylation. Proc Natl Acad Sci U S A.

[B42] Cui J, Rohr LR, Swanson G, Speights VO, Maxwell T, Brothman AR (2001). Hypermethylation of the caveolin-1 gene promoter in prostate cancer. Prostate.

[B43] Toyota M, Ho C, Ahuja N, Jair KW, Li Q, Ohe-Toyota M, Baylin SB, Issa JP (1999). Identification of differentially methylated sequences in colorectal cancer by methylated CpG island amplification. Cancer Res.

[B44] Sasaki M, Tanaka Y, Perinchery G, Dharia A, Kotcherguina I, Fujimoto S, Dahiya R (2002). Methylation and inactivation of estrogen, progesterone, and androgen receptors in prostate cancer. J Natl Cancer Inst.

[B45] Grady WM, Willis J, Guilford PJ, Dunbier AK, Toro TT, Lynch H, Wiesner G, Ferguson K, Eng C, Park JG, Kim SJ, Markowitz S (2000). Methylation of the CDH1 promoter as the second genetic hit in hereditary diffuse gastric cancer. Nat Genet.

[B46] Toyooka S, Toyooka KO, Harada K, Miyajima K, Makarla P, Sathyanarayana UG, Yin J, Sato F, Shivapurkar N, Meltzer SJ, Gazdar AF (2002). Aberrant methylation of the CDH13 (H-cadherin) promoter region in colorectal cancers and adenomas. Cancer Res.

[B47] Muller C, Readhead C, Diederichs S, Idos G, Yang R, Tidow N, Serve H, Berdel WE, Koeffler HP (2000). Methylation of the cyclin A1 promoter correlates with gene silencing in somatic cell lines, while tissue-specific expression of cyclin A1 is methylation independent. Mol Cell Biol.

[B48] Ghoshal K, Majumder S, Li Z, Dong X, Jacob ST (2000). Suppression of metallothionein gene expression in a rat hepatoma because of promoter-specific DNA methylation. J Biol Chem.

[B49] Viswanathan M, Tsuchida N, Shanmugam G (2003). Promoter hypermethylation profile of tumor-associated genes p16, p15, hMLH1, MGMT and E-cadherin in oral squamous cell carcinoma. Int J Cancer.

[B50] Fearon ER (2000). BRCA1 and E-cadherin promoter hypermethylation and gene inactivation in cancer-association or mechanism?. J Natl Cancer Inst.

[B51] Soengas MS, Capodieci P, Polsky D, Mora J, Esteller M, Opitz-Araya X, McCombie R, Herman JG, Gerald WL, Lazebnik YA, Cordon-Cardo C, Lowe SW (2001). Inactivation of the apoptosis effector Apaf-1 in malignant melanoma. Nature.

[B52] Rachmilewitz EA (2000). The role of methylation in CML. Przegl Lek.

[B53] Gonzalez-Gomez P, Bello MJ, Alonso ME, Arjona D, Lomas J, de Campos JM, Isla A, Rey JA (2003). CpG island methylation status and mutation analysis of the RB1 gene essential promoter region and protein-binding pocket domain in nervous system tumours. Br J Cancer.

[B54] Fan X, Munoz J, Sanko SG, Castresana JS (2002). PTEN, DMBT1, and p16 alterations in diffusely infiltrating astrocytomas. Int J Oncol.

[B55] Ghimenti C, Fiano V, Chiado-Piat L, Chio A, Cavalla P, Schiffer D (2003). Deregulation of the p14ARF/Mdm2/p53 pathway and G1/S transition in two glioblastoma sets. J Neurooncol.

[B56] Dong S, Pang JC, Hu J, Zhou LF, Ng HK (2002). Transcriptional inactivation of TP73 expression in oligodendroglial tumors. Int J Cancer.

[B57] Loiseau H, Arsaut J, Demotes-Mainard J (1999). p73 gene transcripts in human brain tumors: overexpression and altered splicing in ependymomas. Neurosci Lett.

[B58] Satoh Y, Nakagawachi T, Nakadate H, Kaneko Y, Masaki Z, Mukai T, Soejima H (2003). Significant Reduction of WT1 Gene Expression, Possibly Due to Epigenetic Alteration in Wilms' Tumor. J Biochem (Tokyo).

[B59] Tomita Y, Bilim V, Hara N, Kasahara T, Takahashi K (2003). Role of IRF-1 and caspase-7 in IFN-gamma enhancement of Fas-mediated apoptosis in ACHN renal cell carcinoma cells. Int J Cancer.

[B60] Detjen KM, Murphy D, Welzel M, Farwig K, Wiedenmann B, Rosewicz S (2003). Downregulation of p21(waf/cip-1) mediates apoptosis of human hepatocellular carcinoma cells in response to interferon-gamma. Exp Cell Res.

[B61] Lu R, Au WC, Yeow WS, Hageman N, Pitha PM (2000). Regulation of the promoter activity of interferon regulatory factor-7 gene. Activation by interferon and silencing by hypermethylation. J Biol Chem.

[B62] Esteller M, Risques RA, Toyota M, Capella G, Moreno V, Peinado MA, Baylin SB, Herman JG (2001). Promoter hypermethylation of the DNA repair gene O(6)-methylguanine-DNA methyltransferase is associated with the presence of G:C to A:T transition mutations in p53 in human colorectal tumorigenesis. Cancer Res.

[B63] Whitehall VL, Walsh MD, Young J, Leggett BA, Jass JR (2001). Methylation of O-6-methylguanine DNA methyltransferase characterizes a subset of colorectal cancer with low-level DNA microsatellite instability. Cancer Res.

[B64] Esteller M, Garcia-Foncillas J, Andion E, Goodman SN, Hidalgo OF, Vanaclocha V, Baylin SB, Herman JG (2000). Inactivation of the DNA-repair gene MGMT and the clinical response of gliomas to alkylating agents. N Engl J Med.

[B65] Toyooka KO TS, Virmani AK, Sathyanarayana UG, Euhus DM, Gilcrease M, Minna JD, Gazdar AF (2001). Loss of expression and aberrant methylation of the CDH13 (H-cadherin) gene in breast and lung carcinomas. Cancer Res.

[B66] Azarschab P, Stembalska A, Loncar MB, Pfister M, Sasiadek MM, Blin N (2003). Epigenetic control of E-cadherin (CDH1) by CpG methylation in metastasising laryngeal cancer. Oncol Rep.

[B67] Yamanaka M, Watanabe M, Yamada Y, Takagi A, Murata T, Takahashi H, Suzuki H, Ito H, Tsukino H, Katoh T, Sugimura Y, Shiraishi T (2003). Altered methylation of multiple genes in carcinogenesis of the prostate. Int J Cancer.

[B68] Pfeifer GP, Yoon JH, Liu L, Tommasi S, Wilczynski SP, Dammann R (2002). Methylation of the RASSF1A gene in human cancers. Biol Chem.

[B69] Toyota M, Ohe-Toyota M, Ahuja N, Issa JP (2000). Distinct genetic profiles in colorectal tumors with or without the CpG island methylator phenotype. Proc Natl Acad Sci U S A.

