# Supplementary material for: Methylation profiles of thirty four promoter-CpG islands and concordant methylation behaviours of sixteen genes that may contribute to carcinogenesis of astrocytoma
Source: BMC Cancer. 2004 Sep 14;4:65. doi: 10.1186/1471-2407-4-65 (PMC520749; doi:10.1186/1471-2407-4-65)
Supplement: Additional File 1 — The target promoter CpG islands and the primer pairs for methylation specific PCR. This file contains his study. [file 1471-2407-4-65-S1.pdf]

Additional file 1, The target promoter CpG islands and the primers for MSP

Gene Name	GeneBank No.	Sense 5'-3'	Antisense 5'-3'	Amplicon lication relative to transcription start	Size (bp)
ABL (M)	U07563	CGAGGATCGTTATTGGTTCG	TAACCGAAACGCGCCTATTA	-368 to -172	197
ABL (U)		TTGTTATTGGTTTGGGTTGG	TAACCACAACCACATCTCCAC	-362 to -150	213
APAF1 (M)	AB070829	AGGTTTAGTTACGTTTCGTTCG	CGTCCACTCGCTACCTCTTC	-348 to-99	217
APAF1 (U)		GAGGTTTAGTTATGTTTTGTTTGTGG	CCACTCACTACCTCTTCTTCTCC	-348 to -102	215
APC (M)	U02509	TATTGCGGAGTGCGGGTC	TCGACGAACTCCCGACGA	-17147 to -17050	97
APC (U)		GTGTTTTATTGTGGAGTGTGGGTT	CCAATCAACAAACTCCCAACAA	-17153 to -17046	108
AR (M)	M58158	TTTCGAGATTTCGGGGAG	ACAACTCCGAACGACGAC	-3 to +210	214
AR (U)		TTTTGAGATTTTGGGGAGTTAG	CCAAACAACAACTTCAAAACCA	-3 to +204	208
BRCA1 (M)	L78833	GGTTAATTTAGAGTTTCGAGAGACG	TCAACGAACTCACGCCGCGCAATCG	-320 to -138	182
BRCA1 (U)		GGTTAATTTAGAGTTTTGAGAGATG	TCAACAAACTCACACCACACAATCA	-320 to -138	182
CAV (M)	NT_007933	TTAGGGCGAGAGCGATTC	GCCGCCAAAAATCAAAAC	+1320 to +1482	163
CAV (U)		AGTGTTTAGGGTGAGAGTGATTTG	CCACCCACCACCAAAAAT	+1315 to +1488	174
CDH1 (M)	L34545	GTGGGCGGGTCGTTAGTTTC	CTCACAAATACTTTACAATTCCGACG	-265 to -93	172
CDH1 (U)		GGTGGGTGGGTTGTTAGTTTTGT	AACTCACAAATCTTTACAATTCCAAC	-266 to -93	172
CDH13 (M)	AB001090	TCGCGGGGTTCGTTTTTCGC	GACGTTTTCATTCATACACGCG	-267 to -24	243
CDH13 (U)		TTGTGGGGTTTGTTTTTTGT	AACTTTTCATTCATACACACA	-267 to -24	243
CSPG2(M)	AC026696	AGTTTCGGGGGACGTTT	TTTTCTACCCCGCTCTCC	+1350 to +1525	176
CSPG2(U)		GAGTTTTGGGGGATGTTTTT	AACACCCAAACCACTCCA	+1349 to +1500	152
cyclin a1 (M)	AF124143	TCGTCGCGTTTTAGTCGT	ACCCGTTCTCCCAACAAC	-755 to -550	206
cyclin a1 (U)		GGGTAGTTTTGTTGTGTTTTAGTTG	AACCACTAACAACCCCCTCT	-762 to -565	199
DAPK1 (M)	NM_004938	GGATAGTCGGATCGAGTTAACGTC	CCCTCCCAAACGCCGA	-332 to -234	98
DAPK1 (U)		GGATAGTTGGATTGAGTTAATGTC	CAAATCCCTCCCAAACACCAA	-332 to -229	103
DBCCR1 (M)	AL138894	CGGGTGTAGCGTTTCGTA	CAAAAACCCCCTCCCTAA	+396 to +561	166
DBCCR1(U)		GGTGGTGTTGGGTGTAGTG	CAAAAACCCCCTCCCTAA	+388 to +561	174
EPO (M)	NT_007933	TTTACGTTTCGGCGAGTT	CGAACGACCGAAATAACC	-212 to -28	185
EPO (U)		TTTTGTTTATGTTTTGGTGAGTTTTA	ACCCCAATCCAACTCCAA	-217 to +17	236
hMLH1 (M)	AB017806	ACGTAGACGTTTTATTAGGGTCGC	CCTCATCGTAACTACCCGCG	-531 to -655	124
hMLH1 (U)		TTTTGATGTAGATGTTTTATTAGGGTTGT	ACCACCTCATCATAACTACCCACA	-526 to -654	118
IRF 7(M)	NT_008953.8	GTTTCGCGGAGTTGAGAATC	TATAACCGACGCGCACAC	+161 to +356	196
IRF 7 (U)		GGTGGGGTTTTGTGGAGT	TACAAATATAACCAACACACACACAT	+155 to +362	208
LKB1 (M)	XM_012913	GGTGTTCGTCGGTTCGTA	TTCCGACTTCCCTTCTCC	-253 to -68	168
LKB1(U)		TTTGGTGTTTGTTGGTTTGTA	TATATTCCAACTTCCCTTCTCC	-238 to -64	175
MAGEA1 (M)	U82670	GTTCGGTCGAAGGAATTTGA	CCACAACCCTCCCTCTTAAA	+24 to +345	322
MAGEA1 (U)		GTTTGGTTGAAGGAATTTGA	ACCCACAACCCTCCCTCTTA	+24 to +347	324
MGMT(M)	AL355531	AGCGTCGTTGTTTTGTGC	CGCTTTCAAAACCACTCG	-451 to -266	186
MGMT(U)		TTGGTAGTGTTGTTGTTTTGTGT	CATCCTACAACCCCCACA	-469 to -261	209
MT1A (M)	K01383	TAAGGTTGGGTTTTCGGAAC	AAATACGAACCACGAAACCA	-421 to -258	164
MT1A (U)		TAAGGTTGGGTTTTTGGAAT	CTCCCCTAAATACAAACCACA	-421 to -251	171
MYOD1 (M)	AF027148	GACGGTTTTCGACGGTTT	GCCCGAAACCGAATACAC	+210 to +393	184
MYOD1 (U)		ATTTGATGGTTTTTGATGGTTT	CACACACATACTCATCCTCACA	+206 to +418	213
OCT6 (M)	L26494	ACGAGGATGCGTTTAGTTCG	GAATCGATCTCCTCCAACCA	+727 to +955	229
OCT6 (U)		GGATGAGGATGTGTTTAGTTTG	TCAATCTCCTCCAACCACTT	+725 to +952	228
p14^{ARF} (M)	L41934	GTCGAGTTCGGTTTTGGAGG	AAAACCACAACGACGAACG	+95 to +255	160
p14^{ARF} (U)		TGAGTTTGGTTTTGGAGGTGG	AACCACAACAACAAACACCCCT	+97 to +262	165
p15^{INK4b} (M)	NM_004936	GCGTTCGTATTTTGCGGTT	CGTACAATAACCGAACGACCGA	-312 to -165	147
p15^{INK4b} (U)		TGTGATGTGTTTGTATTTTGTGGTT	CCATACAATAACCAAACAACCAA	-318 to -164	154
p16^{INK4a} (M)	NM_000077	TTATTAGAGGGTGGGGCGGATCGC	ACCCCGAACCGCGACCGTAA	-80 to +69	149
p16^{INK4a} (U)		TTATTAGAGGGTGGGGTGGATTGT	CAACCCCAAACCACAACCATAA	-80 to +71	151
p27^{KIP1} (M)	AB003688	CGACGTCGGTAAGGTTTG	AAACGCGCAAAAACTACG	-355 to -163	193
p27^{KIP1} (U)		TGTGATTTTGATGTTGGTAAGGT	CAAACCACAACCCAAACTCT	-363 to -141	223
p57^{KIP2} (M)	HSU48869	GGGTTCGCGCGTATAAA	ATACGAAAAACGCGACGA	-70 to +118	205
p57^{KIP2} (U)		GGGTGGGGTTGTGTGTAT	AAACACAACAACTACCTAACTATCCA	-79 to +111	203
p73 (M)	AB031234	GCGTTCGGTTCGTAGGTT	CTCAACTCCCAAAACCCAA	-1722 to -1511	212
p73 (U)		TGGGTGTTTGGTTTGTAGGT	CCAACTCTCAACTCCCAAAA	-1725 to -1505	221
PTEN (M)	NM_000314	GGTTTTTCGAGGCGTTCG	CGCCTCACAACGACTCAACT	-978 to -786	192
PTEN (U)		TGGTTTTTTGAGGTGTTTG	TTCCATCATAACTACAACTTCCA	-979 to -812	167
RASSF1A (M)	XM_040961	GTGTTAACGCGTTGCGTATC	AACCCCGCGAACTAAAAACGA	+82 to +176	94
RASSF1A(U)		TTTGGTTGGAGTGTGTTAATGTG	CAAACCCCACAAACTAAAAACAA	+70 to +178	108
RASSF1C(M)	NT_006014.7	AGTTTGGATTGTCGGTTTCG	TCACAAACCCCACCTACCAC	-370 to -137	187
RASSF1C(U)		GGAGTTTGGATTGTTGGTTTTG	CACCCCCAAAAATAACCTCAT	-370 to -137	187
RB1 (M)	L11910	GGGAGTTTCGCGGACGTGAC	ACGTCGAAACACGCCCCG	-90 to 74	163
RB1 (U)		GGGAGTTTTGTGGATGTGAT	ACATCAAAACACACCCCA	-90 to 74	163
SURVIVIN (M)	U75285	GGCGGGAGGATTATAATTTTCG	CCGCCACCTCTACCAACG	-171 to -7	163
SURVIVIN (U)		GGTGGGAGGATTATAATTTTTG	CCACCACCACCACCTCTAC	-171 to -7	163
VHL (M)	AF010238	TGGAGGATTTTTTTGCGTACGC	GAACCGAACGCCGCGAA	-183 to -25	158
VHL (U)		GTTGGAGGATTTTTTTGTGTATGT	CCCAAACCAAACACCACAAA	-185- to -20	165
WT1(M)	X74840	GTTAGGCGTCGTCGAGGTTA	AAAACGCAAAATCCAACACC	+321 to +526	206
WT1 (U)		TGGGATTTGGGTGGTATTTG	CACCAACACCCACTACACCA	+295 to +510	216
