# Supplementary figures and images for: Methylation profiles of thirty four promoter-CpG islands and concordant methylation behaviours of sixteen genes that may contribute to carcinogenesis of astrocytoma
Source: BMC Cancer. 2004 Sep 14;4:65. doi: 10.1186/1471-2407-4-65 (PMC520749; doi:10.1186/1471-2407-4-65)

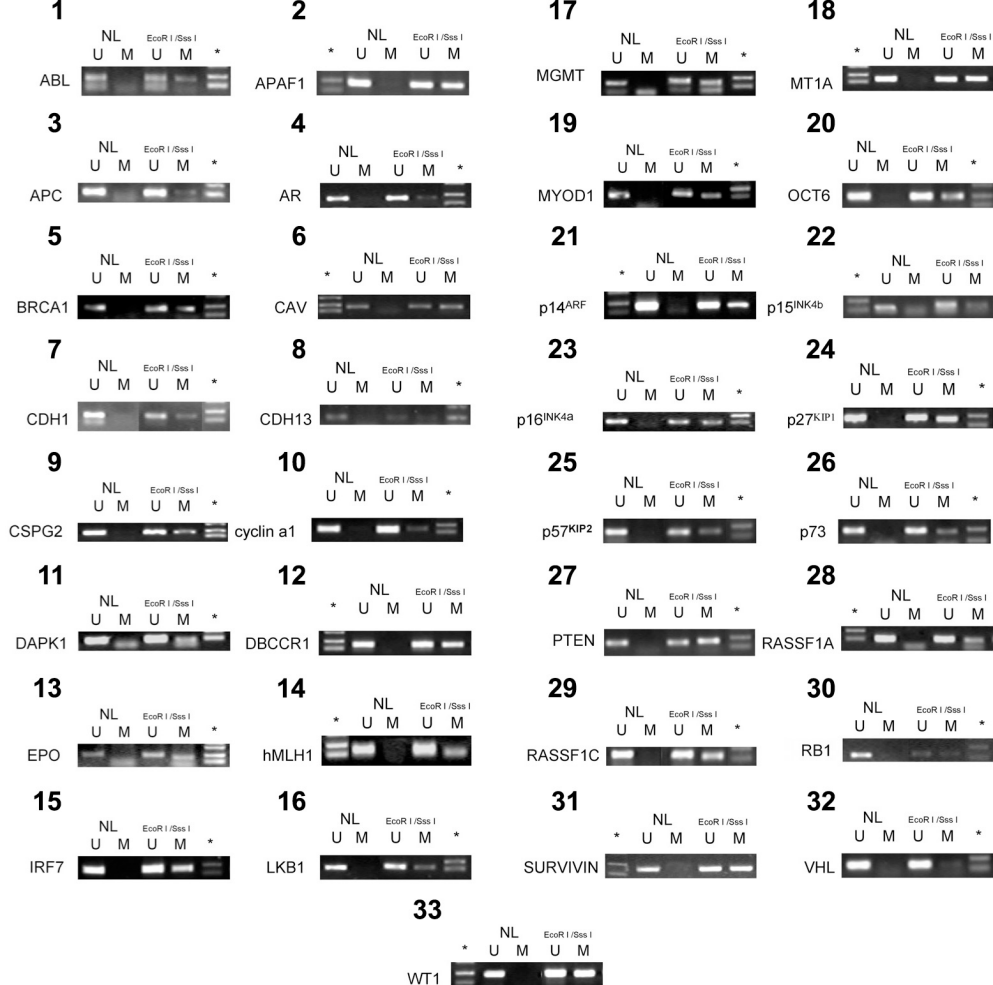

Supplement: Additional File 2 — Methylation profiles of thirty three genes on the in vitro methylated genomic DNA by M. Sss I methyl transferase. The Eco RI restricted genomic DNA from the liver tissue of a healthy donor was in vitro methylated overnight with M. Sss I methyl transferase and subjected to the MSP analysis, followed by electrophoresis in a 1.3% agarose gel. *, the DNA size markers, NL, the untreated sample, U and M, MSP with the pair of primers specific to the unmethylated and methylated targets, respectively. Panels: 1, ABL; 2, APAF1; 3, APC; 4, AR; 5, BRCA1; 6, CAV; 7, CDH1; 8, CDH13; 9, CSPG2; 10, cyclin a1; 11, DAPK1; 12, DBCCR1; 13, EPO; 14, hMLH1; 15, IRF7; 16, LKB1; 17, MGMT; 18, MT1A; 19, MYOD1; 20, OCT6; 21, p14ARF; 22, p15INK4b; 23, p16INK4a; 24, p27 KIP1; 25, p57KIP2; 26, p73; 27, PTEN; 28, RASSF1A; 29, RASSF1C; 30, RB1; 31, SURVIVIN; 32, VHL and 33, WT1. [file 1471-2407-4-65-S2.pdf]

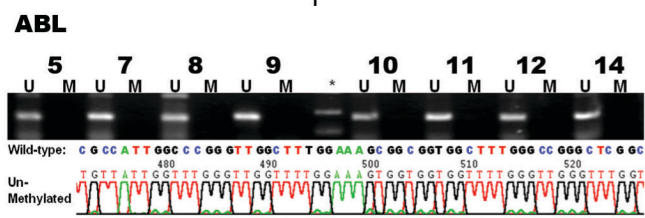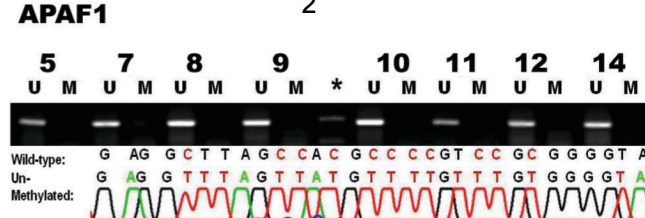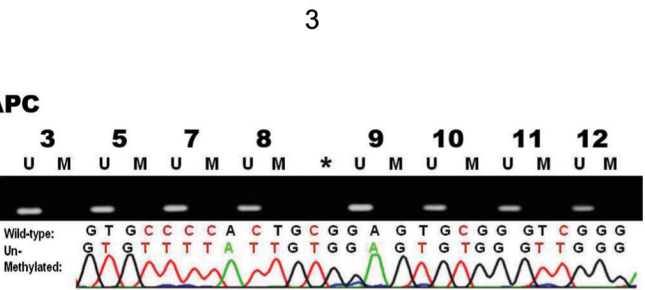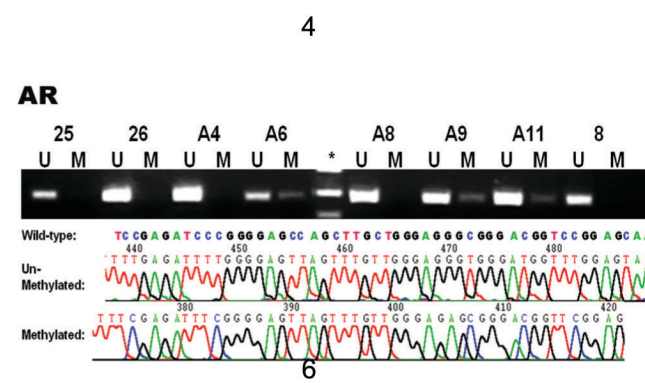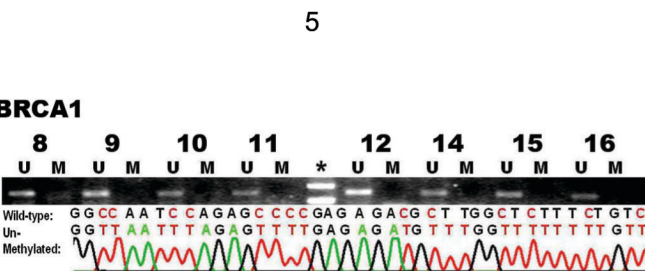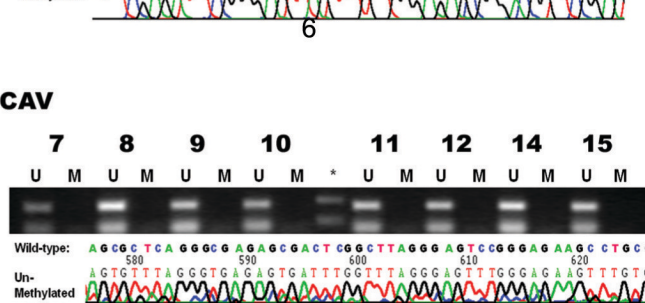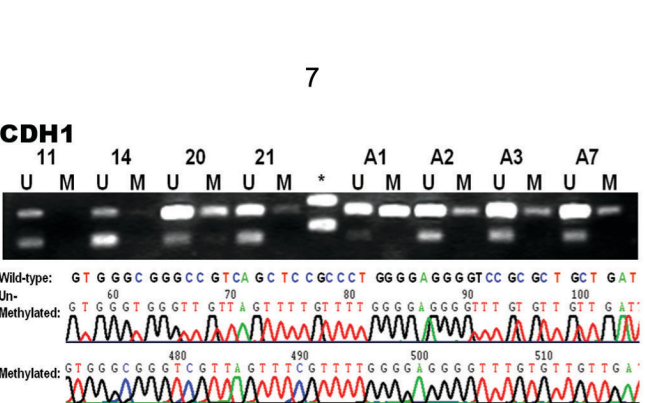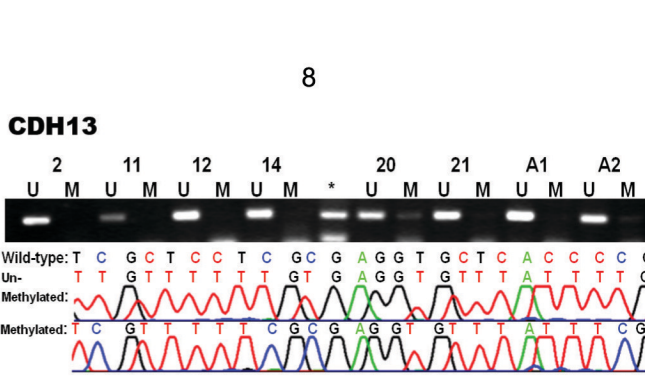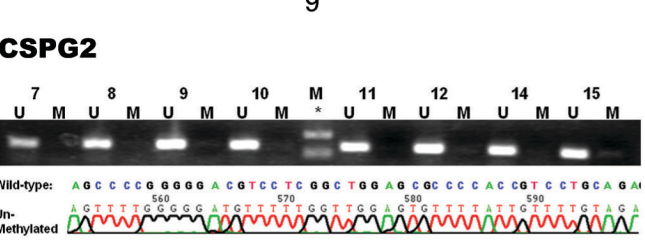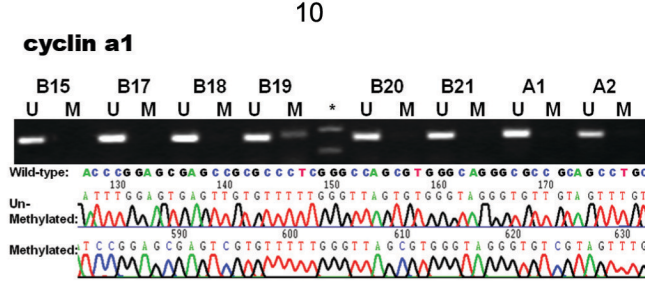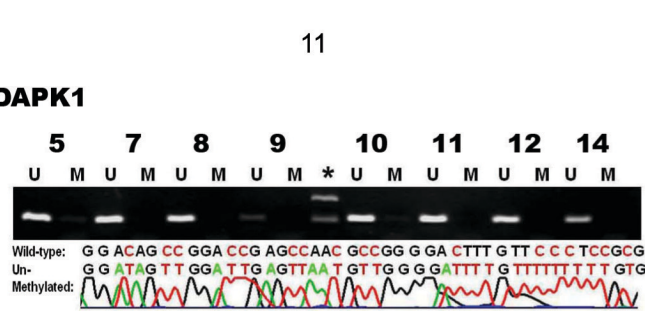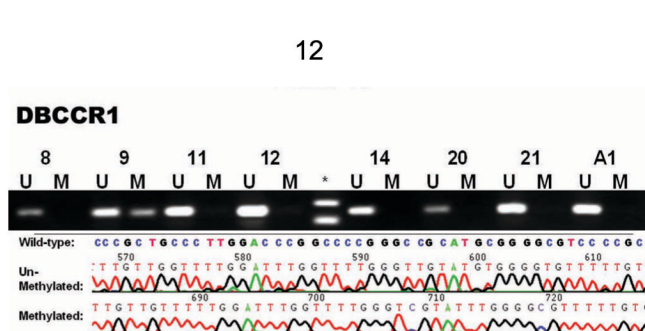

Supplement: Additional File 3 — Methylation profiles of thirty four genes in astrocytoma (part I). Both electrophoretic patterns of the representative PCR products of each of thirty four targets (indicated respectively, at the top of figures) and the sequencing verification of the one representative PCR product were presented. To indicate the methylation status, the sequenced data are aligned with the wild-type sequence. *, size markers, the bands of 250 bp and 100 bp were shown. U, the unmethylated; M, the hypermethylated. Panels: 1, ABL; 2, APAF1; 3, APC; 4, AR; 5, BRCA1; 6, CAV; 7, CDH1; 8, CDH13; 9, CSPG2; 10, cyclin a1; 11, DAPK1 and 12, DBCCR1. [file 1471-2407-4-65-S3.pdf]

## IRF7

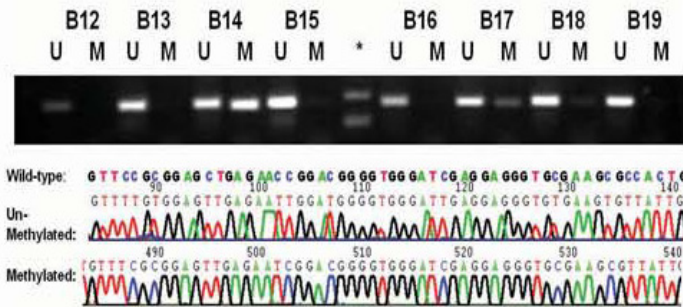

17

## MAGEA1

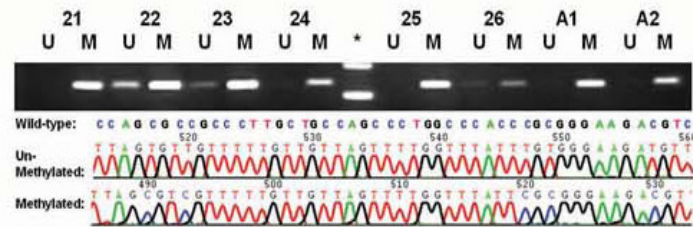

19

**MT1A**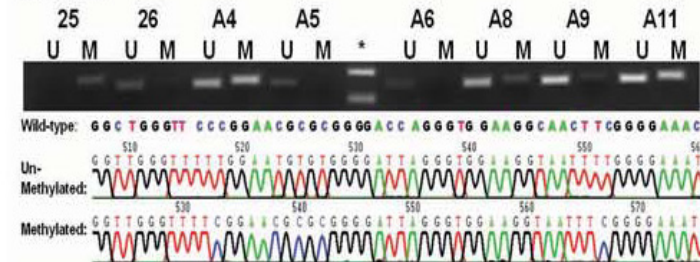

21

**OCT6**

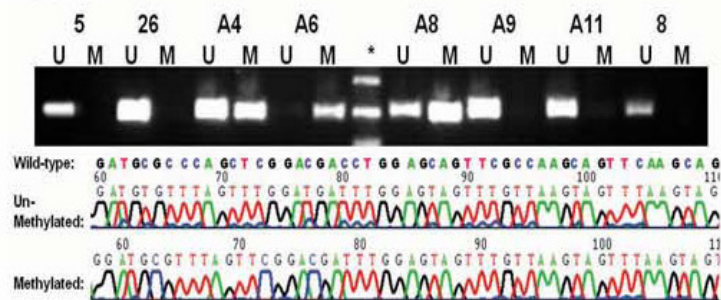

16

**LKB1**

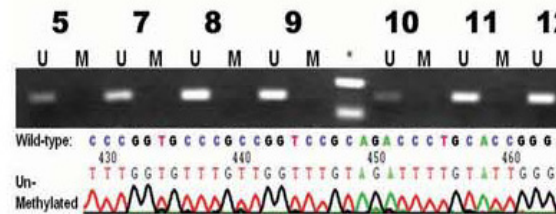

18

## MGMT

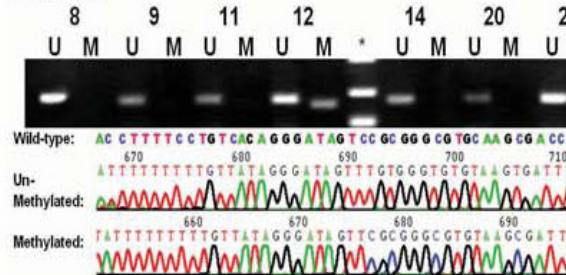

20

## MYOD1

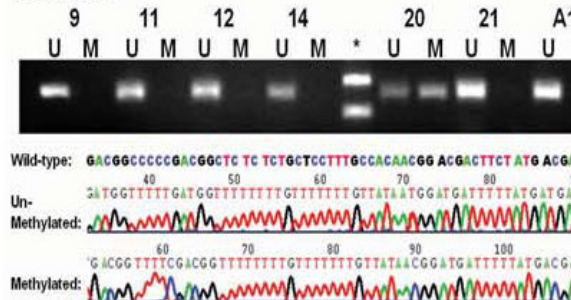

22

**p14<sup>ARF</sup>**

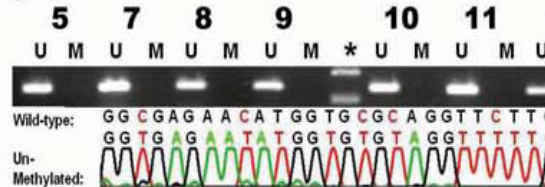

Supplement: Additional File 4 — Methylation profiles of the promoter CpG islands of thirty four genes in astrocytoma (part II). Both electrophoretic patterns of the representative PCR products of each of thirty four targets (indicated respectively, at the top of figures) and the sequencing verification of the one representative PCR product were presented. To indicate the methylation status, the sequenced data are aligned with the wild-type sequence. *, size markers, the bands of 250 bp and 100 bp were shown. U, the unmethylated; M, the hypermethylated. Panels: 13, EPO; 14, hMLH1; 15, IRF7; 16, LKB1; 17, MAGEA1; 18, MGMT; 19, MT1A; 20, MYOD1; 21, OCT6 and 22, p14ARF>. [file 1471-2407-4-65-S4.pdf]

25

p27<sup>KIP1</sup>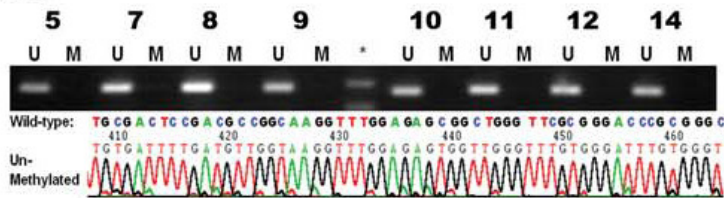

Methylated:

26

p57<sup>KIP2</sup>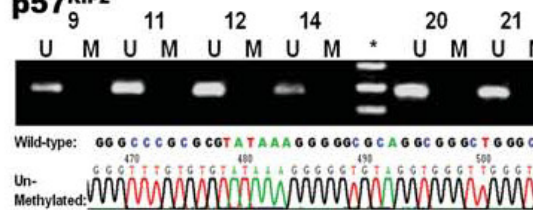

p73

27

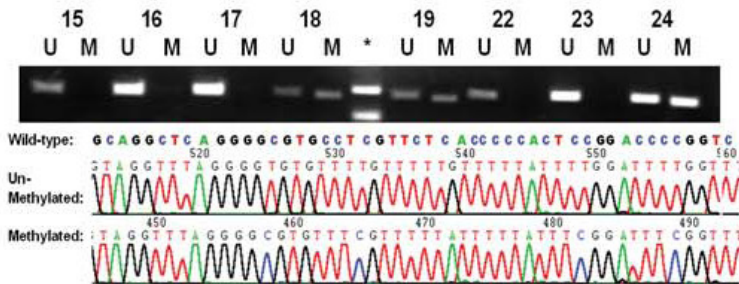

28

PTEN

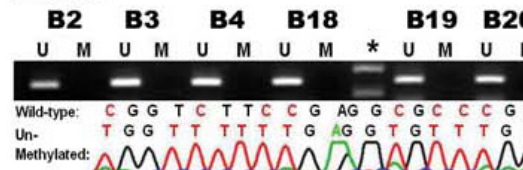

RASSF1A

29

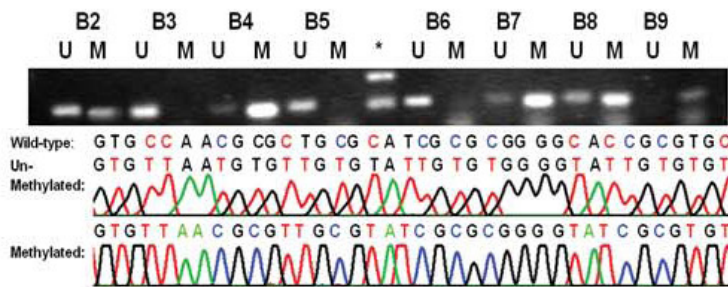

RASSF1C

30

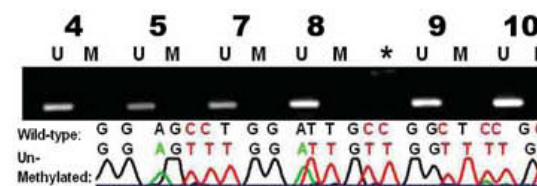

31

RB1

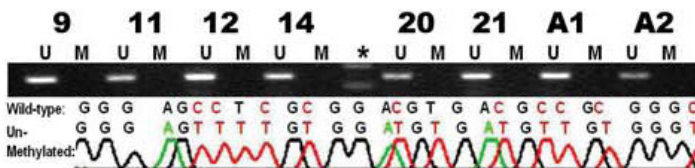

32

SURVIVIN

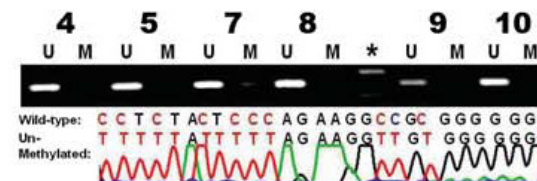

VHL

33

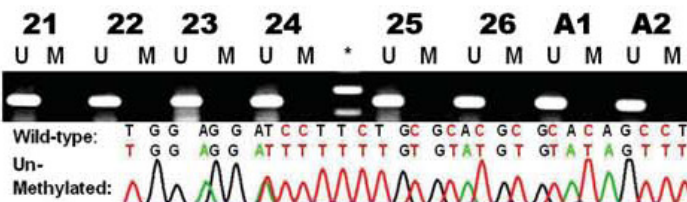

34

WT1

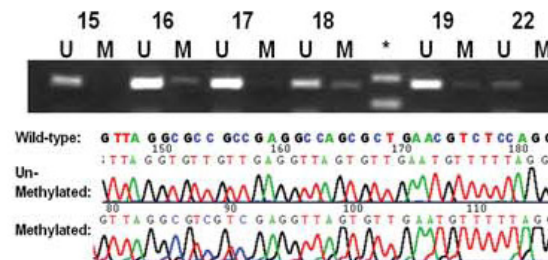

Supplement: Additional File 5 — Methylation profiles of the promoter CpG islands of thirty four genes in astrocytoma (part III). Both electrophoretic patterns of the representative PCR products of each of thirty four targets (indicated respectively, at the top of figures) and the sequencing verification of the one representative PCR product were presented. To indicate the methylation status, the sequenced data are aligned with the wild-type sequence. *, size markers, the bands of 250 bp and 100 bp were shown. U, the unmethylated; M, the hypermethylated. Panels: 23, p15INK4b; 24, p16INK4a; 25, p27 KIP1; 26, p57KIP2; 27, p73; 28, PTEN; 29, RASSF1A; 30, RASSF1C; 31, RB1; 32, SURVIVIN; 33, VHL and 34, WT1. [file 1471-2407-4-65-S5.pdf]

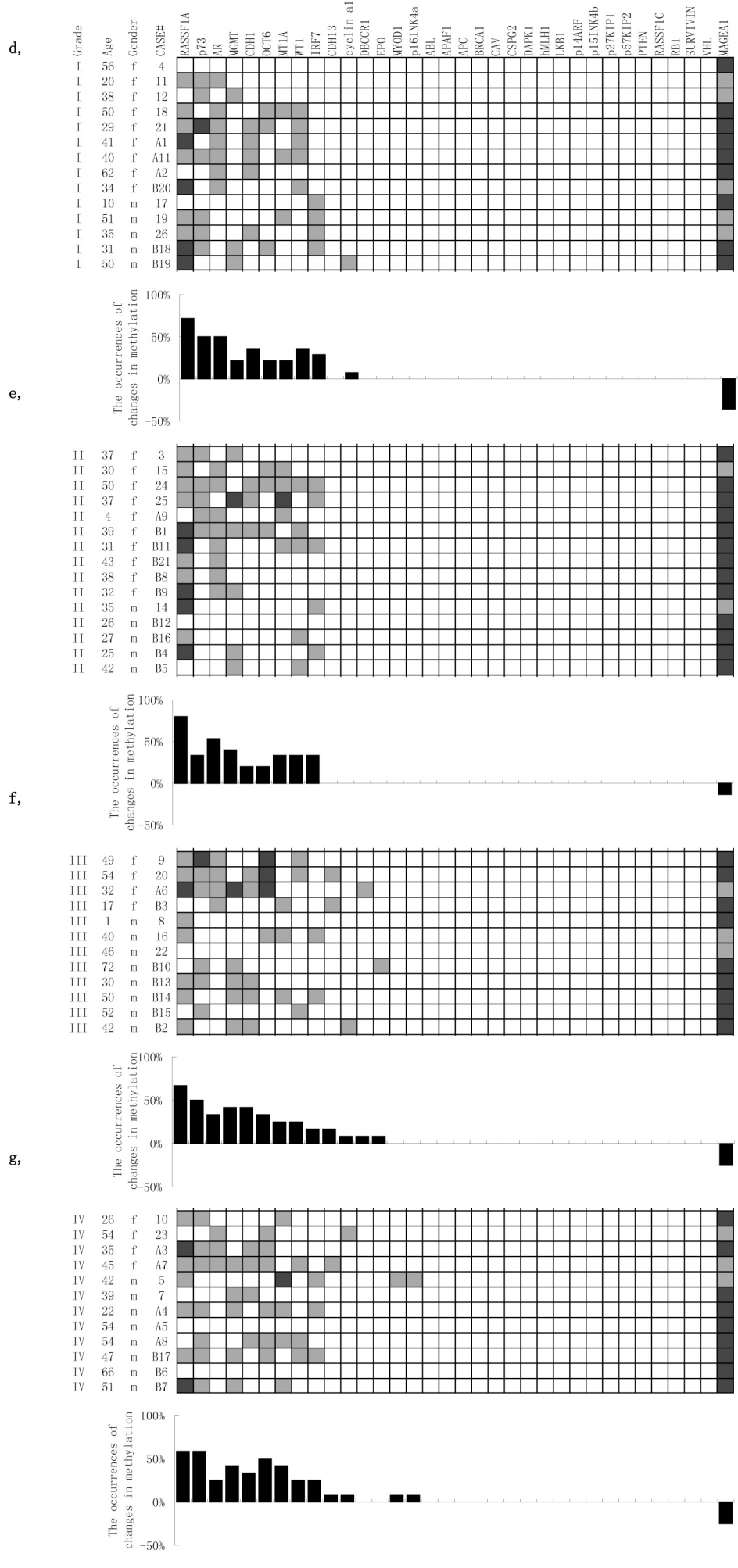

Supplement: Additional File 7 — The summary of the astrocytoma cases displaying no or changes in the methylation profiles (part II). The frequency (%) of the astrocytoma displaying no or the changes in the methylation profile of each target from the normal control were counted and presented in table as well as plotted in the figure below. Sub-tables d-h, the WHO grading I to IV, respectively; The filled, shading and empty boxes indicate the cases where only hypermethylated allele, both hypermethylated and unmethylated alleles and only unmethylated alleles were detected, respectively. The frequency (%) of the hypermethylated targets (except for the MAGEA1, where the heterozygously hypermethylated) among the total cases was presented in the plot. [file 1471-2407-4-65-S7.pdf]
