# Supplementary material for: Methylation profiles of thirty four promoter-CpG islands and concordant methylation behaviours of sixteen genes that may contribute to carcinogenesis of astrocytoma
Source: BMC Cancer. 2004 Sep 14;4:65. doi: 10.1186/1471-2407-4-65 (PMC520749; doi:10.1186/1471-2407-4-65)
Supplement: Additional File 6 — The summary of the astrocytoma cases displaying no or changes in the methylation profiles (part I). The frequency (%) of the astrocytoma displaying no or the changes in the methylation profile of each target from the normal control were counted and presented in table as well as plotted in the figure below. The filled, shading and empty boxes indicate the cases where only hypermethylated allele, both hypermethylated and unmethylated alleles and only unmethylated alleles were detected, respectively. The frequency (%) of the hypermethylated targets (except for the MAGEA1 gene) among the total cases was scored for positive changes in astrocytoma. The MAGEA1 was fully methylated (3/3, 100%) in the control, and become partially demethylated in some astrocytoma, therefore, demethylation of the MAGEA1 in astrocytoma was scored as positive changes. Sub-tables: a, the female patient group, b, the male patient group, and c, the control. [file 1471-2407-4-65-S6.pdf]

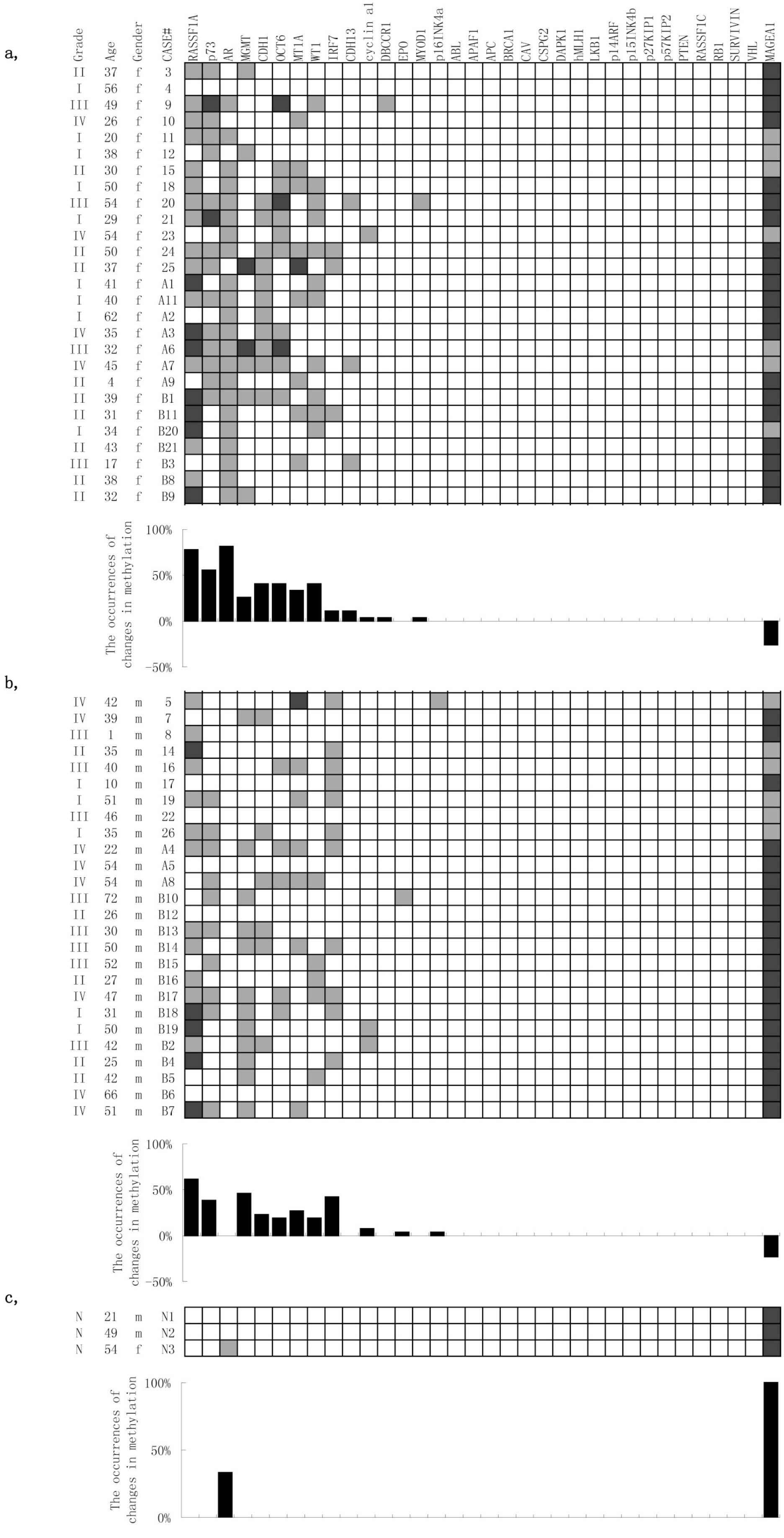
a,
b,
c,
Grade
Age
Gender
CASE#
RASSF1A
p73
AR
MGMT
CDH1
OCT6
MT1A
WT1
IRF7
CDH13
cyclin a1
DBCCR1
EPO
MYOD1
p16INK4a
ABL
APAF1
APC
BRCA1
CAV
CSPG2
DAPK1
hMLH1
LKB1
p14ARF
p15INK4b
p27KIP1
p57KIP2
PTEN
RASSF1C
RB1
SURVIVIN
VHL
MAGEA1
The occurrences of changes in methylation
100%
50%
0%
-50%
