# Supplementary material for: Methylation profiles of thirty four promoter-CpG islands and concordant methylation behaviours of sixteen genes that may contribute to carcinogenesis of astrocytoma
Source: BMC Cancer. 2004 Sep 14;4:65. doi: 10.1186/1471-2407-4-65 (PMC520749; doi:10.1186/1471-2407-4-65)
Supplement: Additional File 8 — The occurrences and frequency of changes in methylation. *, One of three cases was methylated; **, The MAGEA1 gene was fully methylated in the normal tissues and partially demethylated in astrocytoma patients as indicated in the relevant cells. Therefore, the astrocytoma associated changes in methylation of this gene is opposite to the rest, i.e., demethylation rather than hypermethylation. Figure is each cells are the frequency in % and occurrence (case number). [file 1471-2407-4-65-S8.pdf]

	Grade I (14)	Grade II (15)	Grade III (12)	Grade IV (12)	Total cases (53)	Normal Tissue (3)
RASSF1A	71.43(10)	80.00(12)	66.67(8)	58.33(7)	69.81(37)	0.00(0)
p73	50.00(7)	33.33(5)	50.00(6)	58.33(7)	47.17(25)	0.00(0)
AR	50.00(7)	53.33(8)	33.33(4)	25.00(3)	41.51(22)	33.33(1)*
MGMT	21.43(3)	40.00(6)	41.67(5)	41.67(5)	35.85(19)	0.00(0)
CDH1	35.71(5)	20.00(3)	41.67(5)	33.33(4)	32.08(17)	0.00(0)
OCT6	21.43(3)	20.00(4)	33.33(4)	50.00(6)	30.19(16)	0.00(0)
MT1A	21.43(3)	33.33(5)	25.00(3)	41.67(5)	30.19(16)	0.00(0)
WT1	35.71(5)	33.33(5)	25.00(3)	25.00(3)	30.19(16)	0.00(0)
IRF7	28.57(4)	33.33(5)	16.67(2)	25.00(3)	26.42(14)	0.00(0)
CDH13	0.00(0)	0.00(0)	16.67(2)	8.33(1)	5.66(3)	0.00(0)
cyclin a1	7.14(1)	0.00(0)	8.33(1)	8.33(1)	5.66(3)	0.00(0)
DBCCR1	0.00(0)	0.00(0)	8.33(1)	0.00(0)	1.89(1)	0.00(0)
EPO	0.00(0)	0.00(0)	8.33(1)	0.00(0)	1.89(1)	0.00(0)
MYOD1	0.00(0)	0.00(0)	0.00(0)	8.33(1)	1.89(1)	0.00(0)
p16INK4a	0.00(0)	0.00(0)	0.00(0)	8.33(1)	1.89(1)	0.00(0)
ABL	0.00(0)	0.00(0)	0.00(0)	0.00(0)	0.00(0)	0.00(0)
APAF1	0.00(0)	0.00(0)	0.00(0)	0.00(0)	0.00(0)	0.00(0)
APC	0.00(0)	0.00(0)	0.00(0)	0.00(0)	0.00(0)	0.00(0)
BRCA1	0.00(0)	0.00(0)	0.00(0)	0.00(0)	0.00(0)	0.00(0)
CAV	0.00(0)	0.00(0)	0.00(0)	0.00(0)	0.00(0)	0.00(0)
CSPG2	0.00(0)	0.00(0)	0.00(0)	0.00(0)	0.00(0)	0.00(0)
DAPK1	0.00(0)	0.00(0)	0.00(0)	0.00(0)	0.00(0)	0.00(0)
hMLH1	0.00(0)	0.00(0)	0.00(0)	0.00(0)	0.00(0)	0.00(0)
LKB1	0.00(0)	0.00(0)	0.00(0)	0.00(0)	0.00(0)	0.00(0)
p14ARF	0.00(0)	0.00(0)	0.00(0)	0.00(0)	0.00(0)	0.00(0)
p15INK4b	0.00(0)	0.00(0)	0.00(0)	0.00(0)	0.00(0)	0.00(0)
p27KIP1	0.00(0)	0.00(0)	0.00(0)	0.00(0)	0.00(0)	0.00(0)
p57KIP2	0.00(0)	0.00(0)	0.00(0)	0.00(0)	0.00(0)	0.00(0)
PTEN	0.00(0)	0.00(0)	0.00(0)	0.00(0)	0.00(0)	0.00(0)
RASSF1C	0.00(0)	0.00(0)	0.00(0)	0.00(0)	0.00(0)	0.00(0)
RB1	0.00(0)	0.00(0)	0.00(0)	0.00(0)	0.00(0)	0.00(0)
SURVIVIN	0.00(0)	0.00(0)	0.00(0)	0.00(0)	0.00(0)	0.00(0)
VHL	0.00(0)	0.00(0)	0.00(0)	0.00(0)	0.00(0)	0.00(0)
MAGEA1	35.71(5)	13.33(2)	25.00(3)	25.00(3)	24.53(13)	100(3)**
