# Supplementary material for: Methylation profiles of thirty four promoter-CpG islands and concordant methylation behaviours of sixteen genes that may contribute to carcinogenesis of astrocytoma
Source: BMC Cancer. 2004 Sep 14;4:65. doi: 10.1186/1471-2407-4-65 (PMC520749; doi:10.1186/1471-2407-4-65)

The occurrences of changes in methylation (cases) %	Number of gene(s)	0	1	2	3	4	5	6	7	8	9
	Astrocytoma (53)	7.55 (4)	5.66 (3)	13.21 (7)	18.87 (10)	13.21 (7)	20.75 (11)	11.32 (6)	3.77 (2)	3.77 (2)	1.89 (1)
	Normal Tissue (3)	66.67 (2)	33.33 (1)	0.00 (0)	0.00 (0)	0.00 (0)	0.00 (0)	0.00 (0)	0.00 (0)	0.00 (0)	0.00 (1)
	Gene(s) at least	0	1	2	3	4	5	6	7	8	9
	Astrocytoma (53)	7.55 (4)	92.45 (49)	86.79 (46)	73.58 (39)	54.72 (29)	41.51 (22)	20.75 (11)	9.43 (5)	5.66 (3)	1.89 (1)
	Normal Tissue (3)	66.67 (2)	33.33 (1)	0.00 (0)	0.00 (0)	0.00 (0)	0.00 (0)	0.00 (0)	0.00 (0)	0.00 (0)	0.00 (0)

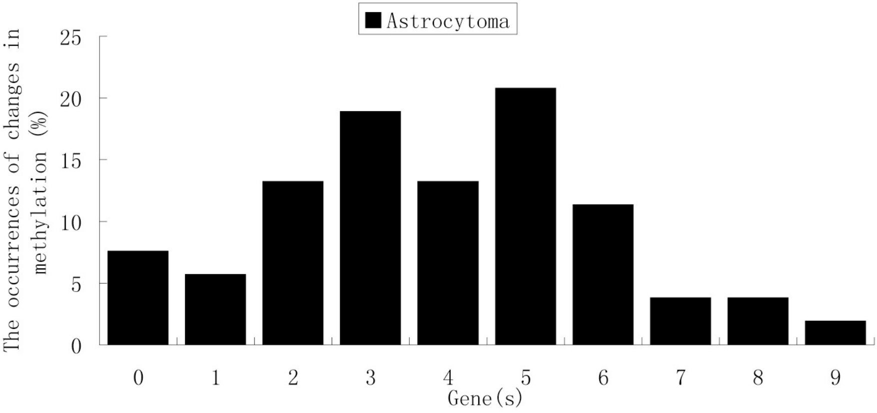

Supplement: Additional File 9 — The summary of changes in the methylation pattern in subsets. Both occurrence (case number) and frequency (%) for the subsets having no change in methylation and changes in one to nine genes are presented in % and (case number) in the top half of table, which was also plotted. Both occurrence (case number) and frequency (%) for the subsets having no change in methylation and changes in, at least, one to nine genes are presented in % and (case number) in the bottom half of table. [file 1471-2407-4-65-S9.pdf]
