# Supplementary material for: Methylation profiles of thirty four promoter-CpG islands and concordant methylation behaviours of sixteen genes that may contribute to carcinogenesis of astrocytoma
Source: BMC Cancer. 2004 Sep 14;4:65. doi: 10.1186/1471-2407-4-65 (PMC520749; doi:10.1186/1471-2407-4-65)
Supplement: Additional File 10 — The summary of the concordant methylation behavior of the hypermethylated targets in astrocytoma. The co-occurrence (/total case) and frequency (%) of a panel subsets consisting of one to three targets were treated with method "Discovery Association Rules" and presented. Sub-tables: a, the total, b, the female, c, the male, and d-g, the grade I to IV, respectively. Column 1 is the number of target in each subset. Column 2 is the co-occurrence (case number/total) (frequency in %). Column 3 is the occurrence of any single target in each subsets, presented in case number (frequency %). The column 4 is the gene(s) in subset. N.B., In view of the strong female inclination of the AR methylation and lacking of any association with astrocytoma, AR has been taken off from this analyses. [file 1471-2407-4-65-S10.pdf]

Total

a,

Number	Co-occurrence(/53)(Frequency)(%)	Occurrence of any target in subset(Frequency)	The gene(s) in subset		
1	37(69.8)	37(69.8)	RASSF1A		
2	20(37.7)	42(79.3)	RASSF1A	p73	
3	11(20.8)	44(83)	RASSF1A	p73	CDH1
3	11(20.8)	43(81.1)	RASSF1A	p73	OCT6

Gender

b, Female

Number	Co-occurrence(/27)(Frequency)(%)	Occurrence of any target in subset(Frequency)	The gene(s) in subset		
1	21(77.8)	21(77.8)	RASSF1A		
2	13(48.1)	23(85.2)	RASSF1A	p73	
3	9(33.3)	25(92.6)	RASSF1A	p73	CDH1

c, Male

Number	Co-occurrence(/26)(Frequency)(%)	Occurrence of any target in subset(Frequency)	The gene(s) in subset		
1	16(61.5)	16(61.5)	RASSF1A		
2	10(38.5)	17(65.4)	RASSF1A	IRF7	
3	5(19.2)	20(76.9)	RASSF1A	IRF7	p73
3	5(19.2)	20(76.9)	RASSF1A	IRF7	MGMT
3	5(19.2)	21(80.8)	RASSF1A	p73	MGMT
3	5(19.2)	18(69.2)	RASSF1A	IRF7	MAGEA1
3	5(19.2)	18(69.2)	RASSF1A	IRF7	MT1A

Grade

d, Grade I

Number	Co-occurrence(/14)(Frequency)(%)	Occurrence of any target in subset(Frequency)	The gene(s) in subset		
1	10(71.4)	10(71.4)	RASSF1A		
2	6(42.9)	11(78.6)	RASSF1A	p73	
3	3(21.4)	12(85.7)	RASSF1A	p73	CDH1
3	3(21.4)	11(78.6)	RASSF1A	p73	MAGEA1
3	3(21.4)	11(78.6)	RASSF1A	CDH1	WT1
3	3(21.4)	12(85.7)	RASSF1A	p73	IRF7

e, Grade II

Number	Co-occurrence(/15)(Frequency)(%)	Occurrence of any target in subset(Frequency)	The gene(s) in subset		
1	12(80.0)	12(80.0)	RASSF1A		
2	5(33.3)	13(86.7)	RASSF1A	MGMT	
2	5(33.3)	12(80.0)	RASSF1A	IRF7	
3	3(20.0)	14(93.3)	RASSF1A	MGMT	p73
3	3(20.0)	13(86.7)	RASSF1A	p73	CDH1
3	3(20.0)	13(86.7)	RASSF1A	IRF7	MT1A

f, Grade III

Number	Co-occurrence(/12)(Frequency)(%)	Occurrence of any target in subset(Frequency)	The gene(s) in subset		
1	8(66.7)	8(66.7)	RASSF1A		
2	5(41.7)	8(66.7)	RASSF1A	CDH1	
3	4(33.3)	9(75.0)	RASSF1A	CDH1	MGMT

g, Grade IV

Number	Co-occurrence(/12)(Frequency)(%)	Occurrence of any target in subset(Frequency)	The gene(s) in subset		
1	7(58.3)	7(58.3)	RASSF1A		
1	7(58.3)	7(58.3)	p73		
2	6(50.0)	8(66.7)	RASSF1A	p73	
3	4(33.3)	9(75.0)	RASSF1A	p73	MGMT
3	4(33.3)	9(75.0)	RASSF1A	p73	OCT6
